# Enzymatic lipid oxidation by eosinophils propagates coagulation, hemostasis, and thrombotic disease

**DOI:** 10.1084/jem.20161070

**Published:** 2017-07-03

**Authors:** Stefan Uderhardt, Jochen A. Ackermann, Tobias Fillep, Victoria J. Hammond, Johann Willeit, Peter Santer, Manuel Mayr, Markus Biburger, Meike Miller, Katie R. Zellner, Konstantin Stark, Alexander Zarbock, Jan Rossaint, Irene Schubert, Dirk Mielenz, Barbara Dietel, Dorette Raaz-Schrauder, Cihan Ay, Thomas Gremmel, Johannes Thaler, Christian Heim, Martin Herrmann, Peter W. Collins, Gernot Schabbauer, Nigel Mackman, David Voehringer, Jerry L. Nadler, James J. Lee, Steffen Massberg, Manfred Rauh, Stefan Kiechl, Georg Schett, Valerie B. O’Donnell, Gerhard Krönke

**Affiliations:** 1 Department of Internal Medicine 3 – Rheumatology and Immunology, Friedrich-Alexander-University Erlangen-Nürnberg (FAU) and Universitätsklinikum Erlangen, Erlangen, Germany; 2 Department of Internal Medicine 3, Division of Molecular Immunology, Friedrich-Alexander-University Erlangen-Nürnberg (FAU) and Universitätsklinikum Erlangen, Erlangen, Germany; 3 Department of Cardiology and Angiology, Friedrich-Alexander-University Erlangen-Nürnberg (FAU) and Universitätsklinikum Erlangen, Erlangen, Germany; 4 Department of Cardiac Surgery, Friedrich-Alexander-University Erlangen-Nürnberg (FAU) and Universitätsklinikum Erlangen, Erlangen, Germany; 5 Department of Pediatrics, Friedrich-Alexander-University Erlangen-Nürnberg (FAU) and Universitätsklinikum Erlangen, Erlangen, Germany; 6 Nikolaus Fiebiger Center of Molecular Medicine, Friedrich-Alexander-University Erlangen-Nürnberg (FAU) and Universitätsklinikum Erlangen, Erlangen, Germany; 7 Department of Infection Biology, Institute for Clinical Microbiology, Immunology, and Hygiene, Friedrich-Alexander-University Erlangen-Nürnberg (FAU) and Universitätsklinikum Erlangen, Erlangen, Germany; 8 Systems Immunity Research Institute, School of Medicine, Cardiff University, Cardiff, Wales, UK; 9 Institute of Infection and Immunity, School of Medicine, Cardiff University, Cardiff, Wales, UK; 10 Department of Neurology, Medical University of Innsbruck, Innsbruck, Austria; 11 Bruneck Hospital, Bruneck, Italy; 12 King's British Heart Foundation Centre, Kings College, London, England, UK; 13 Department of Biology, Institute of Genetics, Friedrich-Alexander-University Erlangen-Nürnberg (FAU), Erlangen, Germany; 14 Medizinische Klinik und Poliklinik I, Klinikum der Universität, Ludwig-Maximilians-Universität, Munich, Germany; 15 Department of Biochemistry and Molecular Biology, Division of Pulmonary Medicine, Mayo Clinic in Arizona, Scottsdale, AZ; 16 Department of Anaesthesiology, Intensive Care, and Pain Medicine, University Hospital Münster, Münster, Germany; 17 Department of Medicine I, Clinical Division of Haematology and Haemostaseology, Medical University of Vienna, Vienna, Austria; 18 Department of Internal Medicine II, Division of Angiology, Medical University of Vienna, Vienna, Austria; 19 Institute for Physiology, Center for Physiology and Pharmacology, Medical University of Vienna, Vienna, Austria; 20 Department Medicine, University of North Carolina, Chapel Hill, NC; 21 Department of Internal Medicine, Eastern Virginia Medical School, Norfolk, VA

## Abstract

Blood coagulation is essential for physiological hemostasis but simultaneously contributes to thrombotic disease. However, molecular and cellular events controlling initiation and propagation of coagulation are still incompletely understood. In this study, we demonstrate an unexpected role of eosinophils during plasmatic coagulation, hemostasis, and thrombosis. Using a large-scale epidemiological approach, we identified eosinophil cationic protein as an independent and predictive risk factor for thrombotic events in humans. Concurrent experiments showed that eosinophils contributed to intravascular thrombosis by exhibiting a strong endogenous thrombin-generation capacity that relied on the enzymatic generation and active provision of a procoagulant phospholipid surface enriched in 12/15-lipoxygenase–derived hydroxyeicosatetraenoic acid–phosphatidylethanolamines. Our findings reveal a previously unrecognized role of eosinophils and enzymatic lipid oxidation as regulatory elements that facilitate both hemostasis and thrombosis in response to vascular injury, thus identifying promising new targets for the treatment of thrombotic disease.

## Abstract (precis)

Uderhardt et al. show that eosinophils accumulate in freshly formed thrombi, where they provide a procoagulant surface that is rich in oxidized phospholipids and allows assembly and activation of plasmatic coagulation factors. This mechanism stabilizes the thrombus and enables hemostasis but also contributes to thrombotic disease.

## Introduction

The coagulation cascade represents an evolutionary highly conserved process that enables efficient hemostasis in response to vascular injury. However, an aberrant intravascular activation of the coagulation cascade causes thrombotic disease, including myocardial infarction, ischemic stroke, or deep-venous thrombosis, and thus represents the leading cause of death worldwide (Stevens et al., 2009). Both hemostasis and thrombosis involve the initial formation of a yet unstable platelet aggregate at the site of vascular injury (Wolberg, 2007). The subsequent activation of plasmatic coagulation results in the formation and polymerization of fibrin, an insoluble protein that allows growth and stabilization of the developing thrombus. Initiation of the coagulation cascade essentially relies on the presence of a procoagulant phospholipid surface and the glycoprotein tissue factor (TF; Nemerson, 1968). These two components initiate the sequential assembly and activation of the membrane-associated prothrombinase complex (factor Xa [FXa] and factor Va; Nemerson, 1968), which generates thrombin, the serine protease that converts fibrinogen to fibrin, and thereby provides the backbone of the growing thrombus (Wolberg, 2007).

Although all cellular membranes contain abundant amounts of phospholipids, they are usually inert and do not support coagulation. Platelets are considered to serve as a major source of procoagulant phospholipids, as these cells can actively modify their membrane and oxidize and externalize the aminophospholipids phosphatidylethanolamine (PE) and phosphatidylserine (PS; Suzuki et al., 2010; Thomas et al., 2010; Yang et al., 2012; O’Donnell et al., 2014). Although platelets can potentially acquire TF via leukocyte-derived microparticles, they lack endogenous expression of TF (Bouchard et al., 2010). These findings raise the question about the exact mechanisms and cells that initiate the assembly of the prothrombinase complex during the onset of coagulation and the propagation of intravascular thrombosis (Flaumenhaft, 2014).

Here, we report that thrombotic events in humans are associated with an in increase in the eosinophilic activation marker eosinophilic cationic protein (ECP). Subsequent experiments showed that eosinophils were abundantly found in thrombi that formed upon vascular injury in mice, where these cells critically contributed to thrombin formation and thrombus stabilization as well as to physiological hemostasis. Eosinophils exerted a strong endogenous thrombin-generation capacity that relied on the simultaneous expression of TF and the provision of a procoagulant phospholipid surface that was enriched in 12/15-lipoxygenase (12/15-LO)–derived oxidized phospholipids. In accordance, we observed a diminished thrombus formation and defective hemostatic response in mice carrying a global or an eosinophil-specific deletion of 12/15-LO.

## Results

### Elevation of eosinophil activation markers predicts the onset of cardiovascular events

**Figure 1.**
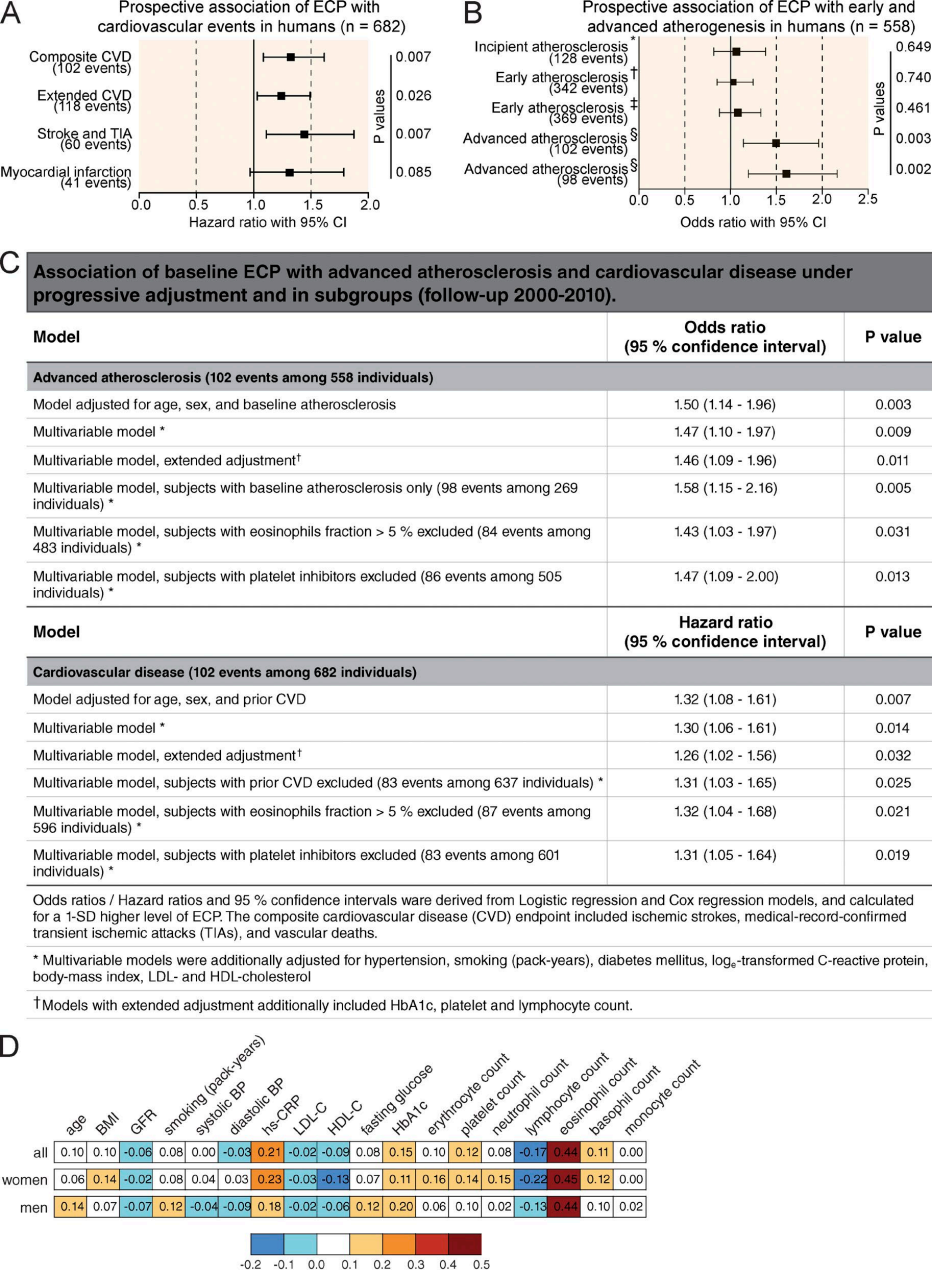
**Elevation of ECP serves as a marker for CVD in humans.** (A and B) Forest plots of the prospective association of baseline ECP with events of CVD (A) and early and advanced atherosclerosis (B) in the Bruneck Study (follow-up 2000 to 2010). All analyses were adjusted for age, sex, and prior CVD. Analyses focusing on ultrasound endpoints were adjusted to the extent of baseline atherosclerosis (log-transformed atherosclerosis summation score). Squares and lines represent hazard ratios of events and 95% confidence intervals (CI). Hazard ratios were derived from Cox regression models and calculated for a 1-SD–higher level of ECP. Composite CVD events considered ischemic strokes, medical record–confirmed TIAs, myocardial infarctions, and vascular deaths. Mean and median follow-ups were 8.6 and 10 yr. The extended CVD events additionally considered revascularization procedures. *, This analysis is confined to 277 individuals free of atherosclerosis at baseline and focused on the manifestation of first carotid plaques. ^†^, This analysis considers all 558 individuals with ultrasound follow-up and focused on the manifestation of new carotid plaques. ^‡^, This analysis considers all 558 individuals with ultrasound follow-up and focused on both the manifestation of new carotid plaques and extension of existing ones. ^§^, This analysis considers all 558 individuals with ultrasound follow-up and focused on the development of advanced complicated plaques (stenosis >40%). The second line is confined to 269 subjects with manifest baseline atherosclerosis. (C) Odds ratios/hazard ratios and 95% confidence intervals derived from logistic regression and Cox regression models and calculated for a 1-SD–higher level of ECP. The composite CVD endpoint included ischemic strokes, medical record–confirmed TIAs, myocardial infarctions, and vascular deaths. *, Multivariable models were additionally adjusted for hypertension, smoking (pack-years), diabetes, log-transformed C-reactive protein, body-mass index, and LDL and HDL cholesterol. ^†^, Models with extended adjustment additionally included HbA1c, platelet, and lymphocyte counts. (D) Correlation pattern of ECP level with blood cell counts and demographic and vascular risk factors in the Bruneck Study (evaluation 2000; *n* = 682 including 354 women and 328 men). BP, blood pressure; GFR, glomerular filtration rate; HbA1c, glycated hemoglobin; HDL-C, HDL cholesterol; hs-CRP, high-sensitivity C-reactive protein; LDL-C, LDL cholesterol. Spearman correlation coefficients are given for all variables. Correlations that are statistically significant after correction for multiple comparisons (Bonferroni-corrected p-value <0.05) are in bold, and corresponding squares have covering colors.

To elucidate mechanisms that trigger thrombotic events during cardiovascular disease (CVD) in humans, we made use of the prospective population-based Bruneck Study (Stegemann et al., 2014). Population characteristics are summarized in Table S1. This study allows unique monitoring of atherosclerosis progression and differentiates between early atherosclerosis, advanced atherosclerotic lesions, and the occurrence of thrombotic events such as stroke and myocardial infarction. In contrast to early atherosclerosis, which primarily reflects a chronic inflammatory intima thickening, both development of advanced atherosclerotic lesions and especially the occurrence of thrombotic events are primarily associated with factors reflecting enhanced plaque vulnerability and a procoagulant state (Willeit et al., 2000). Therefore, we sought to identify novel serologic markers that specifically predict the occurrence of advanced atherosclerotic lesions and thrombotic events. A proteomic proximity ligation assay unexpectedly identified an increased plasma level of ECP as a highly significant predictor of new-onset atherothrombotic events and development of advanced atherosclerotic lesions (Fig. 1, A and B). In contrast, ECP levels were unrelated to early plaque development and growth and showed only weak correlations with age and other vascular risk factors (Fig. 1, B–D). These findings were robust to multivariable adjustment and stable in sensitivity analyses (Fig. 1, C and D). Because plasma ECP levels serve as a potential marker for eosinophil activation, these data were suggestive of a contribution of eosinophils to thrombus formation and vessel occlusion.

### Eosinophils promote intravascular thrombin formation and thrombus growth

**Figure 2.**
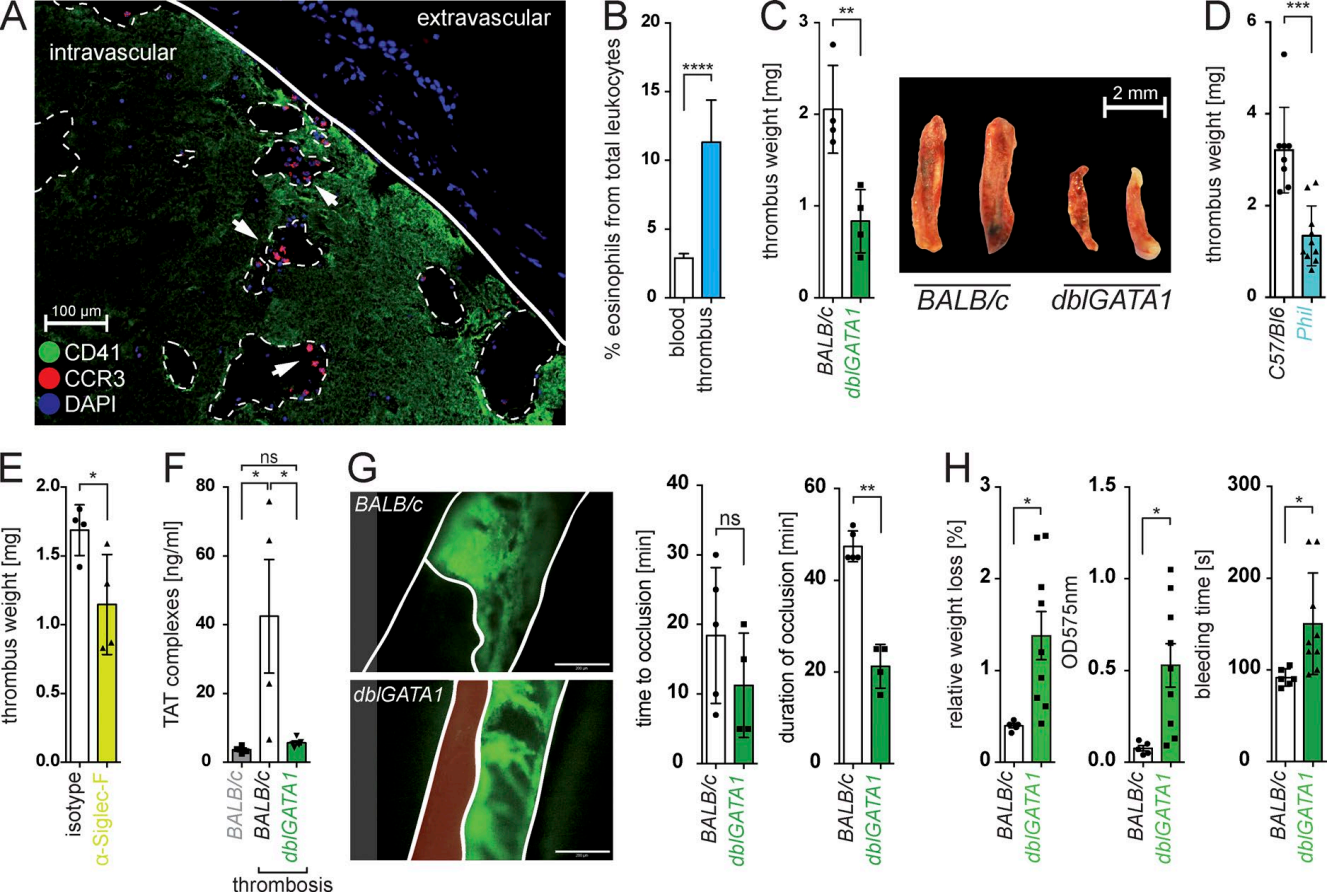
**Eosinophils contribute to intravascular thrombosis and hemostasis.** (A) Representative microscopy image (*n* = 5) showing immunofluorescence staining for platelets (CD41; green), eosinophils (CCR3; red), and cellular nuclei (DAPI; blue) in a ferric chloride (FeCl)–induced thrombus of a mouse IVC. White arrowheads indicate CCR3^+^ eosinophils, and dashed lines mark lacunae surrounded by platelet aggregates. Bar, 100 µm. (B) Relative number of eosinophils in IVC thrombi of WT mice compared with peripheral blood counts. *n* = 5. (C and D) FeCl-induced IVC thrombus of Δ*dblGATA1* mice (BALB/c background; *n* = 4; C), PHIL mice (C57BL/6 background; *n* = 10; D), and their corresponding WT littermates. Bar, 2 mm. (E) FeCl-induced IVC thrombosis in WT mice treated with anti-SiglecF antibody (*n* = 4) or isotope control. (F) Measurement of TAT complexes in plasma from *dblGATA1* mice (*n* = 5) after FeCl-induced IVC thrombosis. (G) Representative image of a FeCl-induced thrombosis of the carotid artery in WT or Δ*dblGATA1* mice (30 min after FeCl-induced injury) and quantification of the kinetics of thrombus formation and dissolution (right; see also supplementary videos). *n* = 4. Bars, 200 µm. (H) Bleeding assays (15-mm tail cut) with WT (*BALB/c*; *n* = 5) and Δ*dblGATA1* mice (*n* = 9). Bar graphs show relative weight loss, OD_575nm_ of the lost blood after lysis, and primary bleeding time (time until the first stop of bleeding). Data are representative of at least three independent experiments. Error bars represent SEM. *, P < 0.05; **, P < 0.01; ***, P < 0.001; ****, P < 0.0001; Student’s *t* test.

Immunofluorescence microscopy of experimentally induced thrombi indeed confirmed enrichment of eosinophils, which accumulated at the boarder of platelet-rich areas throughout the thrombus (Fig. 2, A and B; and Fig. S1, A–E). To determine whether eosinophils actively contributed to the formation of an intravascular thrombus, we consequently performed an injury-induced venous thrombosis model of the inferior venae cava (IVC) in two different eosinophil-deficient mouse strains, namely Δ*dblGATA1* mice (Yu et al., 2002) and PHIL mice (Lee et al., 2004), as well as in their WT littermates. Absence of eosinophils resulted in a dramatically attenuated thrombus formation (Fig. 2, C and D), which supported a functional contribution of these cells. Also, WT mice that had received an eosinophil-depleting anti-SiglecF antibody showed a significantly decreased thrombotic potential (Fig. 2 E and Fig. S1 F).

As these data pointed toward a so far unrecognized role of eosinophils during the initiation and/or propagation of thrombosis, we sought to elucidate the underlying mechanisms. We did not observe alterations in platelet numbers or platelet function or any intrinsic defects of clotting factors in eosinophil-deficient mice (Fig. S2, A–C). However, measurement of plasmatic thrombin–antithrombin (TAT) complexes as a quantitative marker for thrombin generation showed that TAT complexes rapidly appeared after induction of thrombosis in WT mice but were almost absent in eosinophil-deficient mice (Fig. 2 F). These data were indicative of a major contribution of eosinophils to the initiation of plasmatic coagulation and the intravascular generation of thrombin in response to vascular injury. As thrombin-mediated blood coagulation is considered essential for the stabilization of the initial platelet aggregate and thrombus growth (Wolberg, 2007), we consequently used intravital video microscopy to visualize platelet aggregation and thrombus formation upon vascular injury in the mouse carotid artery of WT and eosinophil-deficient mice. Whereas an absence of eosinophils did not impair the formation of a first platelet-rich aggregate at the site of injury, it provoked a rapid dissolution of this yet unstable initial thrombus (Fig. 2 G and Videos 1–4). In line with these findings, we observed an exacerbated blood loss in eosinophil-deficient Δ*dblGATA1* mice after a medium-scale injury (15-mm tail cut; Fig. 2 H), indicative of a defective coagulation-dependent secondary hemostasis. Platelet-dependent primary hemostasis after small-scale injury (3-mm tail cut) was unaffected by the absence of eosinophils (Fig. S2 D). Platelet depletion, in turn, unmasked the defective hemostasis in Δ*dblGATA1* mice after the small-scale injury, which was depicted by a dramatically augmented blood loss (Fig. S2, E and F) and, thus, demonstrated that platelets and eosinophils performed synergistic roles during thrombosis and hemostasis. Notably, absence of eosinophils did not impair thrombus development in the IVC upon flow restriction (Fig. S2 G), suggesting that this leukocyte subset specifically mediated the hemostatic and thrombotic response upon vascular injury but did not contribute to stasis-induced thrombus formation.

### Eosinophils autonomously generate thrombin in a TF-dependent manner

**Figure 3.**
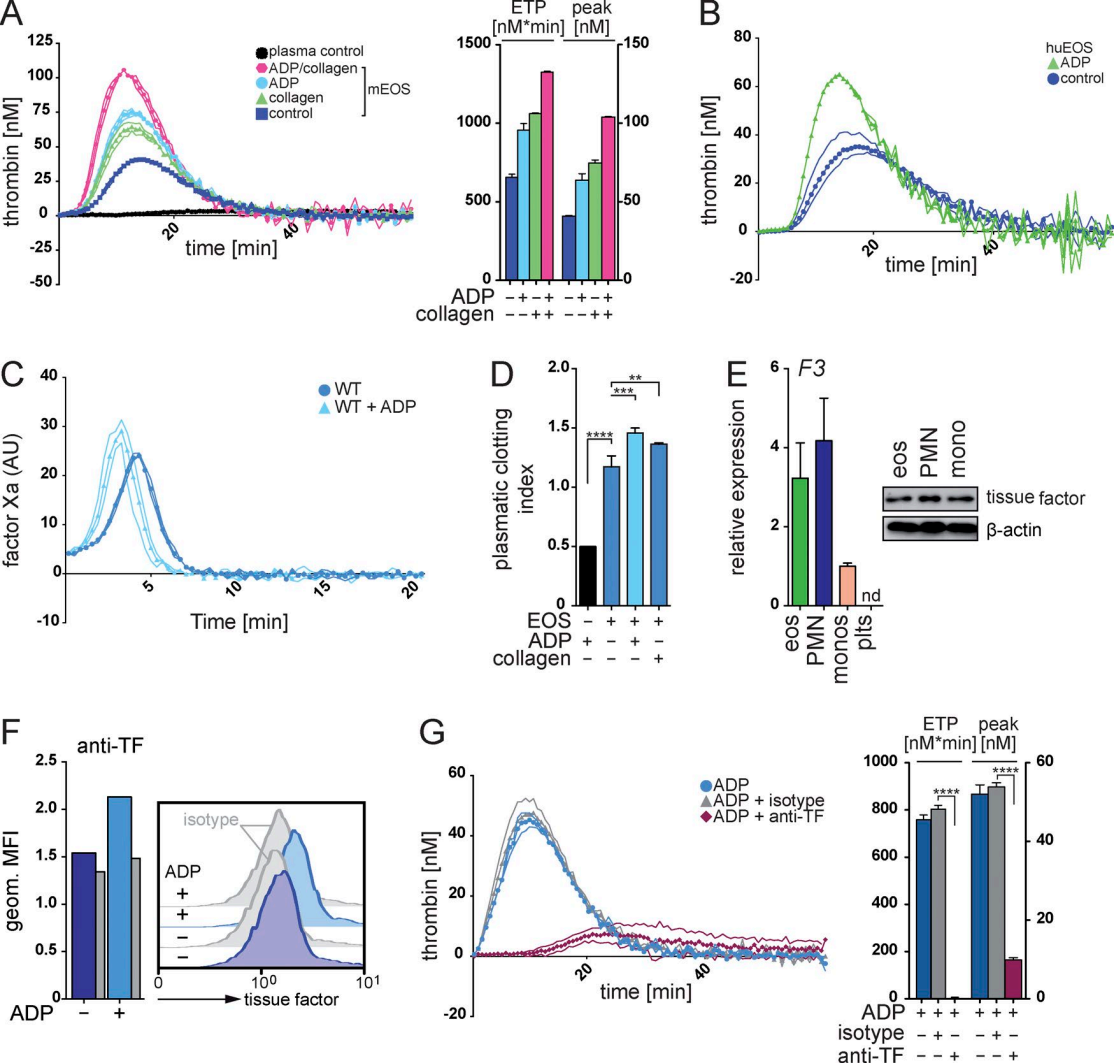
**Eosinophils autonomously initiate thrombin generation.** (A and B) Calibrated thrombin generation curve of in vitro–generated mouse eosinophils (mEOS; A) or human eosinophils (huEOS; B) after stimulation with ADP and/or collagen. Bar graphs show endogenous thrombin potential (ETP; nM*min) and peak of thrombin generation (peak; nM). (C) Calibrated FXa generation curve of mouse eosinophils. AU, arbitrary units. (D) Plasma clotting time experiments with in vitro–generated mouse eosinophils (Eos) stimulated with ADP or collagen. Bar graphs display calculated clotting index. The black bar shows plasma with ADP alone. (E, left) Quantitative RT-PCR analysis of TF mRNA (*F3*) expression in mouse monocytes (monos), neutrophils (PMN), eosinophils (eos), and platelets (plts); expression of the gene of interest was normalized to *Atcb* expression. (Right) Western blot analysis of TF protein (47 kD) expression in sorted mouse leukocytes. (F) Flow cytometry analysis of the exposure of TF on the surface of resting or ADP-stimulated mouse eosinophils. Histograms show representative flow cytometric stainings, and bar graphs show mean geometric fluorescence intensities (geom. MFI). (G, left) Calibrated thrombin generation assay with ADP-stimulated mouse eosinophils in the presence of blocking anti-TF antibody or isotope control. (Right) Bar graphs show endogenous thrombin potential (ETP; nM*min) and peak of thrombin generation (peak; nM). Data are representative of at least three independent experiments. Error bars represent SEM. *, P < 0.05; **, P < 0.01; ***, P < 0.001; ****, P < 0.0001; Student’s *t* test.

Together, our data revealed a key role of eosinophils during injury-induced thrombin generation and blood coagulation in vivo. Consequently, we determined whether these cells were able to autonomously generate thrombin in the absence of exogenous procoagulant phospholipids or recombinant TF. We found that in vitro–differentiated mouse eosinophils as well as isolated human eosinophils indeed exerted a dramatic endogenous thrombin generation potential, which was further augmented by prestimulation of eosinophils with ADP or collagen (Fig. 3, A and B). Thrombin generation was preceded by formation of FXa and followed by the appearance of fibrin clots (Fig. 3, C and D; and Fig. S3 A). These data indicated the involvement of a TF-dependent pathway and suggested that eosinophils were equipped with all the necessary key components for the initiation of plasmatic coagulation, which would include an endogenous provision of TF and procoagulant phospholipids. We confirmed expression of TF by eosinophils, which were additionally able to expose this glycoprotein on their surface upon ADP stimulation (Fig. 3, E and F; and Fig. S3 B). Addition of a blocking antibody against TF abrogated eosinophil-induced thrombin generation, confirming the functional involvement of this glycoprotein (Fig. 3 G).

### Eosinophils actively provide a procoagulant phospholipid surface that supports TF-mediated thrombin generation

**Figure 4.**
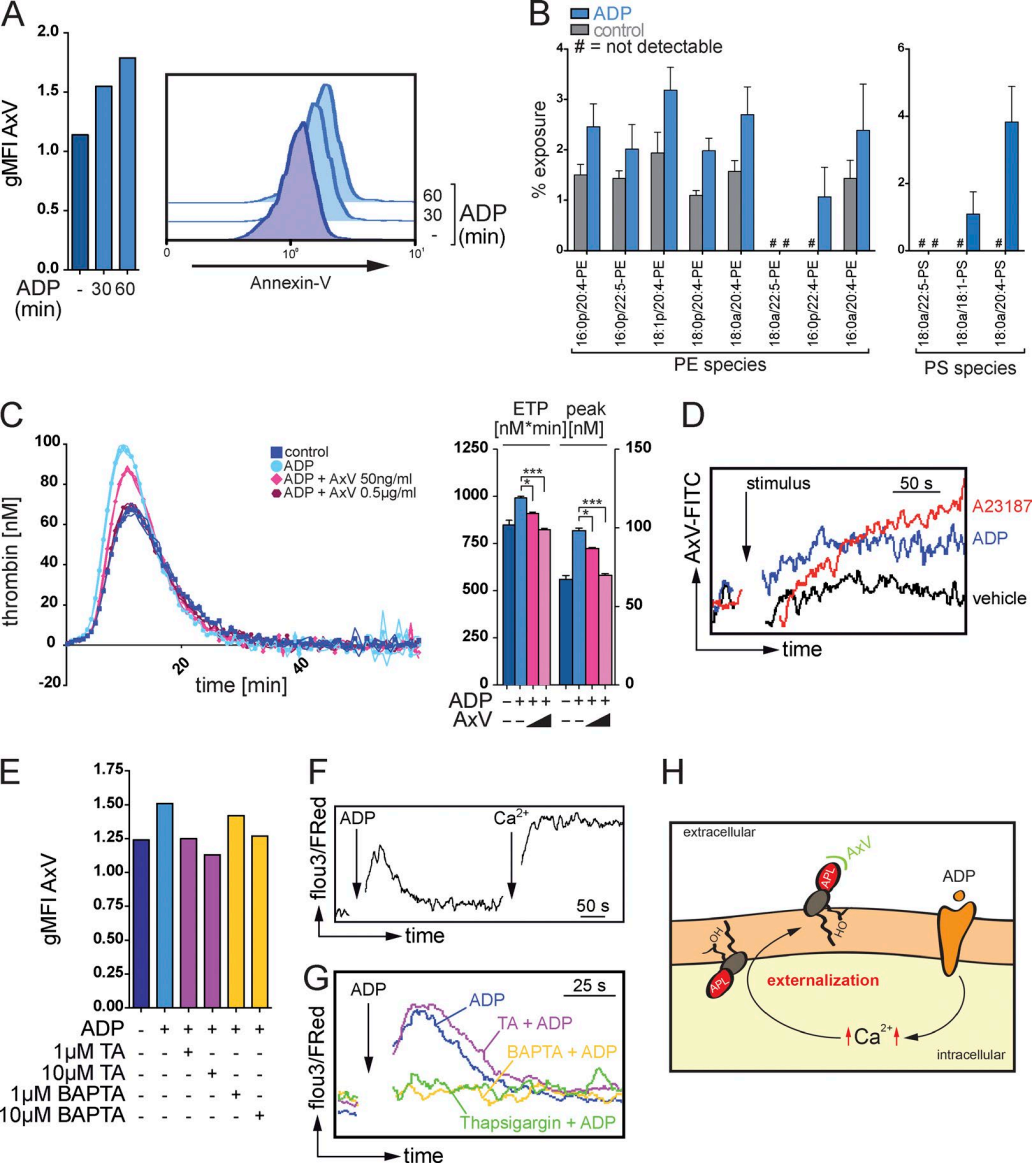
**Ca^2+^-dependent exposure of aminophospholipids by eosinophils promote thrombin generation.** (A) Flow cytometry analysis of the binding of annexin V (AxV) to aminophospholipids on the surface of resting or ADP-stimulated mouse eosinophils. Histograms show representative annexin V stainings, and bar graphs show mean geometric fluorescence intensities (gMFI). (B) LC/MS/MS-based quantification of the exposure of the aminophospholipids PE and PS in mouse eosinophils in response to ADP stimulation. (C, left) Calibrated thrombin generation assays with resting or ADP-stimulated mouse eosinophils in the presence of annexin V. (Right) Bar graphs show endogenous thrombin potential (ETP; nM*min) and peak of thrombin generation (peak; nM). (D) Flow cytometry analysis of annexin V binding on mouse eosinophils over time in the presence of calcium ionophore A23187, ADP, or vehicle. (E) Flow cytometry analysis of annexin V binding on mouse eosinophils in the presence of tannic acid (TA) or intracellular Ca^2+^-chelator BAPTA/AM. Bar graphs show geometric mean fluorescence intensity. (F) Flow cytometry–based analysis of intracellular Ca^2+^ signaling, indicated by Fluo3/FuraRed ratio, over time in a Ca^2+^-free environment. Where indicated (arrow and Ca^2+^), CaCl_2_ at a final concentration of 1 mM was added. (G) Flow cytometry–based analysis of intracellular Ca^2+^ signaling, indicated by Fluo3/FuraRed ratio, over time in a Ca^2+^-free environment. (H) Postulated mechanism of a sequential generation and Ca^2+^-dependent externalization of aminophospholipids (APL) at the surface of eosinophils. OH indicates hydroxyl group. Data are representative of at least three independent experiments. Error bars represent SEM. *, P < 0.05; ***, P < 0.001.

The thrombin generation potential of activated eosinophils was clearly superior to other TF-expressing cells, such as LPS-stimulated macrophages (Fig. S3 C). These findings suggested that additional factors allowed eosinophils to efficiently promote coagulation. As the TF-mediated generation of thrombin is considered to depend on the presence of a procoagulant phospholipid surface (Nemerson, 1968; Mackman, 2004), we determined whether eosinophils underwent specific changes within their plasma membrane that would support plasmatic coagulation. The aminophospholipids PS and PE can facilitate thrombin generation but are usually hidden within the inner leaflet of the plasma membrane and, thus, are unavailable for an interaction with plasmatic coagulation factors (Thomas et al., 2010; Yang et al., 2012). Measurement of annexin V surface binding, a surrogate marker for the exposure of aminophospholipids (Clark et al., 2013), revealed a rapid increase in the annexin V positivity of eosinophils in response to ADP, indicating that these cells actively changed the polarity of their plasma membrane (Fig. 4 A). Exposure of PE and PS species was additionally confirmed using mass spectrometry (Fig. 4 B). However, masking of surface aminophospholipids by annexin V blocked the ADP-induced thrombin generation in a dose-dependent manner (Fig. 4 C), confirming an essential role of these events during eosinophil-induced coagulation.

In platelets, translocation of PS and PE was shown to involve the activity of Ca^2+^-sensitive Cl^−^ channels of the TMEM (transmembrane protein) gene family, which were suggested to additionally control cationic currents and seem to either possess or induce scramblase activity at the plasma membrane (Suzuki et al., 2010; Tian et al., 2012; Yang et al., 2012; Kunzelmann et al., 2014). Also, eosinophils expressed several TMEM family members (Fig. S3 D), and the Ca^2+^ ionophore A23187 rapidly triggered exposure of the aminophospholipids PE and PS in eosinophils (Fig. 4 D and Fig. S3 E). Intracellular chelation of Ca^2+^ by BAPTA/AM or addition of tannic acid, a nonselective inhibitor of the TMEM family of calcium-activated Cl^−^ channels, in turn, interfered with the ADP-induced binding of annexin V to their surface (Fig. 4 E). In line with these findings, ADP stimulation of eosinophils triggered a rapid and transient increase in cytosolic Ca^2+^ levels in a Ca^2+^-free environment, which could be boosted by the addition of extracellular calcium (Fig. 4 F). Prior depletion of intracellular calcium stores by thapsigargin, an inhibitor of the endoplasmic reticulum Ca^2+^ ATPase, blocked the initial ADP-induced increase in cytosolic Ca^2+^ (Fig. 4 G). These data were suggestive of an initial ADP-induced Ca^2+^ release from intracellular stores and a subsequent activation of a store-operated calcium entry through the plasma membrane (Bergmeier et al., 2013) that triggered exposure of procoagulant aminophospholipids to immediately support TF-dependent formation of the prothrombin complex (Fig. 4 H).

### 12/15-LO–mediated formation of procoagulant oxidized phospholipids essentially contributes to the thrombin-generation potential of eosinophils

**Figure 5.**
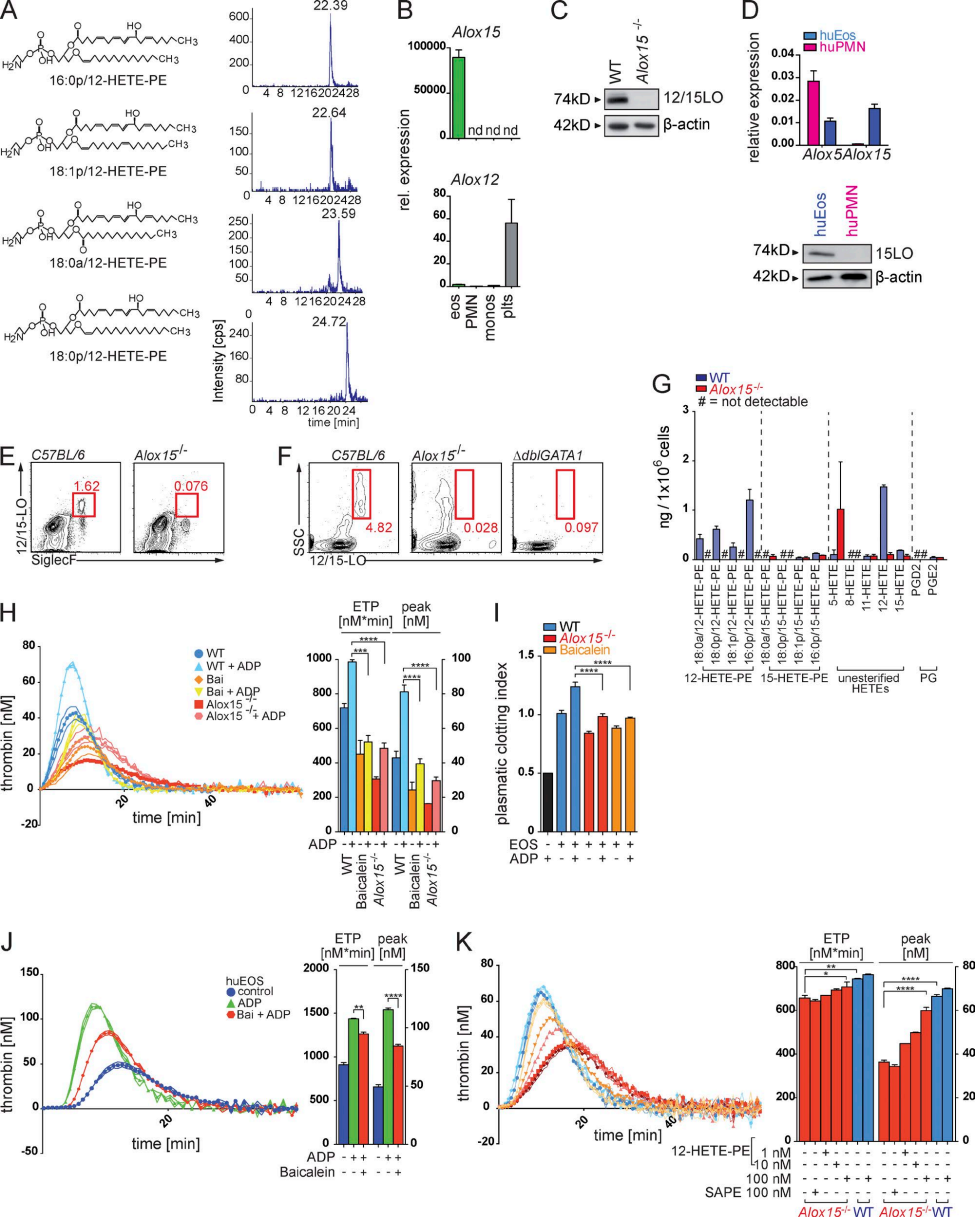
**12/15-LO–mediated oxidation of membrane phospholipids initiates eosinophil-mediated thrombin formation.** (A) Representative LC/MS/MS analysis of different 12-HETE-PE oxidation species in lipid extracts of in vitro–generated mouse eosinophils. Cps, counts per second. (B) Quantitative RT-PCR analysis of 12/15-LO mRNA (*Alox15*) and 12-LO mRNA (*Alox12*) in mouse monocytes (monos; CD11b^+^CD115^+^), neutrophils (PMN; CD11b^+^Ly6G^+^), eosinophils (eos; side scatter^hi^CD11b^+^Siglec-F^+^), and platelets (plts) after FACS. Expression was normalized to *Atcb* expression. rel., relative. (C) Western blot of 12/15-LO protein (74 kD) expression in WT and *Alox15^−/−^* mouse eosinophils. (D) Quantitative RT-PCR analysis of *Alox5* and *Alox15* mRNA (top) and Western blot analysis of 15-LO protein (bottom) expression in human neutrophils (huPMN) and human eosinophils (huEos) isolated by Ficoll density gradient centrifugation and magnetic cell separation. mRNA expression was normalized to *Actb*. (E) Flow cytometry analysis of 12/15-LO in eosinophils (side scatter^hi^SiglecF^+^) isolated from peripheral blood of WT and *Alox15^−/−^* mice. (F) Flow cytometry analysis of the side scatter (SSC)^hi^12/15-LO^+^ population in blood from WT, *Alox15^−/−^*, and Δ*dblGATA1* mice. (G) LC/MS/MS-based quantification of different esterified 12- and 15-HETE-PE, nonesterified 5-, 8-, 11-, 12-, and 15-HETE species, and prostaglandins D2 and E2 (PGD2 and PGE2) in WT (blue) and *Alox15^−/−^* (red) mouse eosinophils. Levels are presented as nanograms per 10^6^ cells. (H, left) Calibrated thrombin generation curve of mouse WT eosinophils, eosinophils treated with baicalein (Bai), and *Alox15^−/−^* eosinophils. (Right) Bar graphs show endogenous thrombin potential (ETP; nM*min) and peak of thrombin generation (peak; nM). (I) Plasmatic clotting time experiments with WT mouse eosinophils (EOS), mouse eosinophils treated with the 12/15-LO inhibitor baicalein, and *Alox15^−/−^* mouse eosinophils. Bar graphs display calculated clotting index. The black bar shows plasma with ADP alone. (J, left) Calibrated thrombin generation assay with lysates generated from human eosinophils in the presence of PRP reagent (see Materials and methods). (Right) Bar graphs show endogenous thrombin potential (nM*min) and peak of thrombin generation (nM). (K, left) Calibrated thrombin generation assays with WT and *Alox15^−/−^* mouse eosinophils in the presence of phosphatidylcholine/PS liposomes carrying oxidized 12-HETE-PE or nonoxidized PE (SAPE) species, as indicated. (Right) Bar graphs show endogenous thrombin potential (nM*min) and peak of thrombin generation (nM). Data are representative of at least three independent experiments. Error bars represent SEM. *, P < 0.05; **, P < 0.01; ***, P < 0.001; ****, P < 0.0001; Student’s *t* test.

Next, we aimed to determine whether specific aminophospholipid species accounted for the increased thrombin-generation potential of eosinophils and performed an additional lipidomic analysis of their phospholipid profile, which showed that their cellular membranes were enriched in a specific set of PE oxidation products (12–hydroxyeicosatetraenoic acid–PEs [12-HETE-PEs]; Fig. 5 A). As oxidation of phospholipids had been suggested to increase their procoagulant potential (Thomas et al., 2010), we sought to determine a potential role of PE oxidation and the different oxidized PE species during eosinophil-mediated thrombin generation. 12-LO (*Alox12*) and 12/15-LO (*Alox15*) are the two major enzymes exerting 12-LO activity in mammals (Kuhn et al., 2015) and thus served as the potential enzymatic source for the identified 12-HETE-PEs in eosinophils. Analysis of peripheral blood leukocytes and platelets showed that *Alox12* was absent in all leukocyte subsets and exclusively expressed in platelets. *Alox15* mRNA and 12/15-LO protein, in turn, were highly expressed in mouse and human eosinophils but absent in platelets, monocytes, neutrophils, and the healthy vascular wall (Fig. 5, B–D; and Fig. S3 F). These data showed that, during the steady state, eosinophils were the only cells expressing *Alox15* within the peripheral blood and the vasculature. 12/15-LO expression was accordingly absent in blood leukocytes of 12/15-LO–deficient (*Alox15^−/−^*) and eosinophil-deficient Δ*dblGATA1* mice (Fig. 5, E and F). Mass spectrometry showed that *Alox15^−/−^* eosinophils indeed lacked the identified 12-HETE-PEs, thus confirming 12/15-LO as the single enzymatic source of these oxidized aminophospholipids in eosinophils (Fig. 5 G).

In accordance with a major contribution of 12/15-LO–mediated membrane oxidation to the thrombin-formation activity of eosinophils, we observed a severely reduced ability of *Alox15^−/−^* eosinophils to generate thrombin and FXa as well as to induce fibrin clots (Fig. 5, H and I; and Fig. S3 G). The 12/15-LO inhibitor baicalein potently interfered with the procoagulant activity of mouse and human eosinophils as well (Fig. 5, H–J), showing that the *Alox15*-mediated control of plasma coagulation by eosinophils represented an evolutionary conserved mechanism and potential target for pharmacologic intervention during thrombotic disease. Of note, 12/15-LO–deficient eosinophils showed no alterations in their eosinophil maturation markers (Fig. S3, H and I). Phospholipase A2 (PLA2)–dependent cleavage of 12/15-LO products from membrane phospholipids was not involved in the 12/15-LO–mediated thrombin generation activity of eosinophils, as inhibition of PLA2 activity did not reduce the procoagulant activity of eosinophils (Fig. S3 J).

We subsequently measured thrombin generation in the presence of liposomes containing different PE species to substitute for 12/15-LO activity. Addition of liposomes with 12-HETE-PE immediately restored the thrombin-generation potential of *Alox15^−/−^* eosinophils, whereas identical liposomes with nonoxidized PEs were ineffective (Fig. 5 K), supporting the concept of a 12/15-LO–mediated control of thrombin generation that was dependent on generation and provision of 12-HETE-PEs.

### 12/15-LO expression in eosinophils promotes thrombotic disease and supports physiological hemostasis

**Figure 6.**
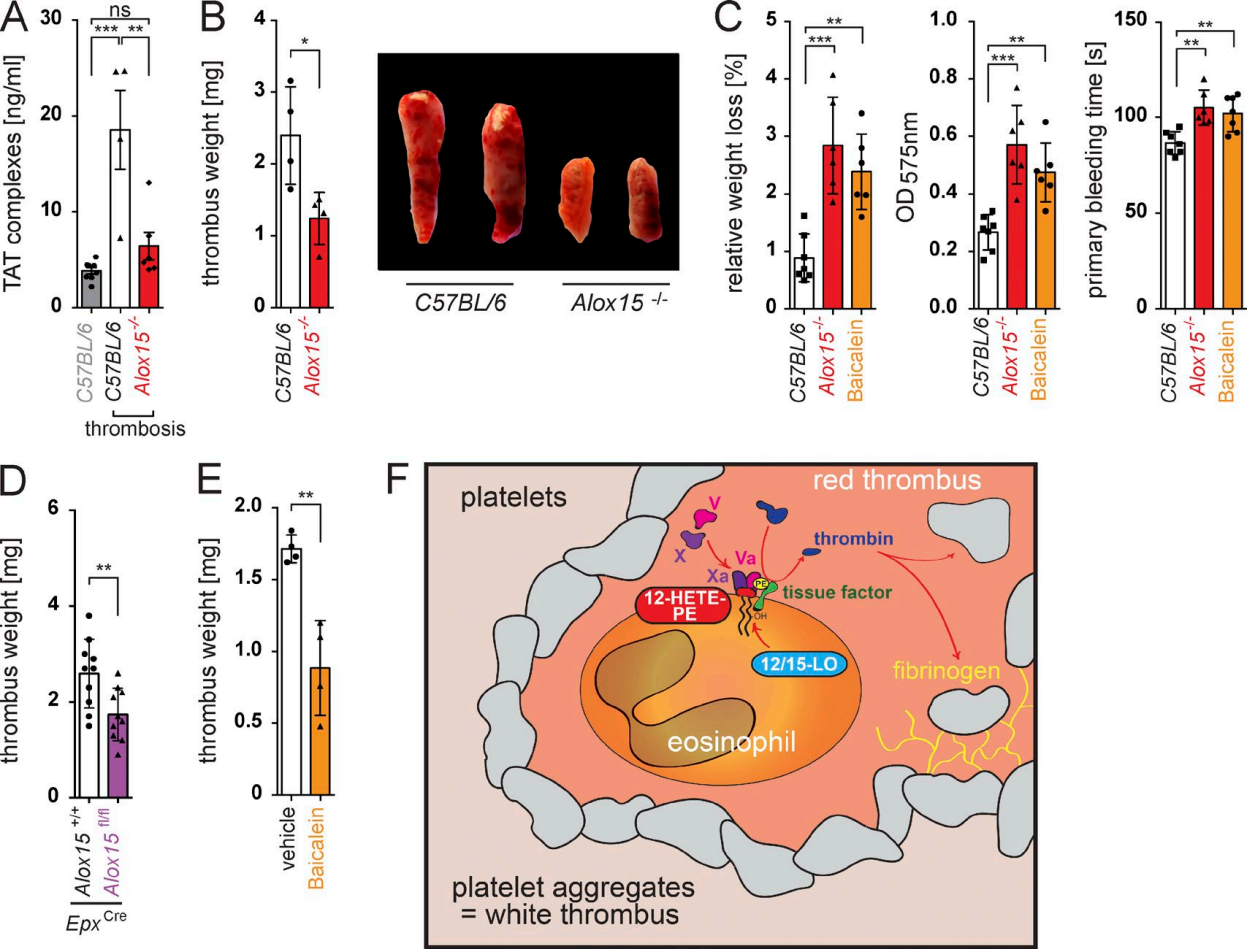
**12/15-LO–induced coagulation contributes to thrombus formation and hemostasis.** (A and B) TAT complex formation (*n* = 6; A) and thrombus formation (*n* = 4; B) after ferric chloride (FeCl)–induced thrombosis of the IVC in *Alox15^−/−^* mice (C57BL/6 background) and WT littermates. (C) Bleeding assays (15-mm tail cut) with WT (*C57BL/6*) mice, *Alox15^−/−^* mice, and WT mice treated with baicalein (*n* = 6 each). Bar graphs show relative weight loss, OD_575nm_ of the lost blood after lysis, and primary bleeding time (time until first stop of bleeding). (D and E) FeCl-induced IVC thrombus formation in mice carrying an eosinophil-specific deletion of *Alox15* (*Alox15^fl/fl^*; *n* = 10) and their WT littermates (*Alox15^+/+^*; *n* = 9; D) and mice that received the 12/15-LO inhibitor baicalein (*n* = 4) and a vehicle (*n* = 4; E). Data are representative of at least three independent experiments. Error bars represent SEM. *, P < 0.05; **, P < 0.01; ***, P < 0.001; Student’s *t* test. (F) Proposed mechanism of the eosinophil-mediated and 12/15-LO–induced thrombin generation in response to platelet aggregation.

To determine the in vivo relevance of 12/15-LO–mediated membrane oxidation by eosinophils during thrombotic disease, we performed an injury-induced thrombosis model in *Alox15^−/−^* mice and their WT littermates. In line with our previous data, absence of 12/15-LO resulted in reduced TAT complex formation (Fig. 6 A) and an impaired thrombus formation (Fig. 6 B). *Alox15^−/−^* mice accordingly displayed a defective hemostatic response (Fig. 6 C), although these animals showed no alterations in the function and number of platelets or in plasmatic coagulation factors (Fig. S4, A–C). We subsequently confirmed a reduced thrombotic potential of mice carrying a conditional deletion of *Alox15* in eosinophils as well as in animals that had received baicalein, demonstrating a key role of the identified pathway during coagulation in vivo and highlighting its potential as a therapeutic target (Fig. 6, D and E).

## Discussion

Until recently, eosinophils were solely envisaged as driving force of parasite defense and as mediators of allergic disease (Jacobsen et al., 2012). However, an increasing amount of data suggests a broader and rather homeostatic role of these cells during the resolution of inflammation and regulation of tissue repair (Rothenberg and Hogan, 2006; Lee et al., 2010; Heredia et al., 2013; Tani et al., 2014). More recently, eosinophils were also described to accumulate in human thrombi (Riegger et al., 2016) and reported to express TF (Moosbauer et al., 2007). Moreover, activated eosinophils were shown to tether to immobilized platelets under shear flow (McCarty et al., 2003), a mechanism which might explain their homing to the freshly formed and yet instable thrombus. Clinical data show that hypereosinophilic patients indeed suffer from an increased incidence of atypical thrombotic events (Ames et al., 2010; Todd et al., 2014). Although these findings have indicated a potential role of this leukocyte subset during thrombus formation, functional evidence that supports a direct contribution of eosinophils to thrombosis or hemostasis as well as insights into involved molecular pathways have been lacking so far. Our current data show that eosinophils critically contribute to thrombin formation and plasmatic coagulation, a process that is often regarded as the most primitive form of immune response to tissue injury and infection (Engelmann and Massberg, 2013). Other leukocyte subsets have been previously implicated as potential sources of TF and might likewise contribute to thrombus formation in certain settings (von Brühl et al., 2012; Engelmann and Massberg, 2013). However, neutrophils and monocytes in the peripheral blood seem to be unable to provide an enzymatically engineered, procoagulant phospholipid surface, whereas platelets express prothrombotic phospholipids but lack intrinsic TF expression. Thus, eosinophils seem to combine the abilities of platelets to oxidize and expose distinct prothrombotic aminophospholipids with the capacity of leukocytes to provide TF within a single cell (Fig. 6 F). Notably, neutrophils were shown to contribute to stasis-induced thrombosis but were fully dispensable during hemostasis after vascular injury (Engelmann and Massberg, 2013). Eosinophils, in turn, seem to primarily mediate the hemostatic and thrombotic response that follows tissue injury, suggesting that different leukocyte subsets fulfill distinct tasks during hemostasis and thrombosis.

On a molecular level, we identify mouse 12/15-LO and its human orthologue 15-LO as key enzymes during the generation of procoagulant phospholipids in eosinophils and show that this pathway directly contributes to the initiation of thrombus formation after injury. Although 12/15-LO expression in macrophage-derived foam cells has been previously implicated in the oxidation of low-density lipoprotein (LDL) particles and the formation of atherosclerotic plaques (Cyrus et al., 2001; Uderhardt and Krönke, 2012), the physiological role of 12/15-LO–mediated lipid oxidation has remained unclear (Kühn and O’Donnell, 2006). Our findings suggest that 12/15-LO–mediated enzymatic lipid oxidation serves as a key mechanism controlling eosinophil-directed coagulation and physiological hemostasis. Thus, our current data provide an explanation for the stringent conservation of this enzymatic pathway during mammalian evolution and simultaneously suggest that eosinophils as well as 12/15-LO are promising new targets for an anticoagulant therapy.

## Material and methods

### Patient cohorts

#### Bruneck Study population

The Bruneck Study is a prospective population-based survey on the epidemiology and pathogenesis of atherosclerosis that started in 1990. The study population was recruited as a sex- and age-stratified random sample of all residents age 40 to 79 living in Bruneck (*n* = 4,739), Northern Italy. Detailed follow up examinations including high-resolution ultrasound of the carotid vessels were performed every 5 yr. The present analysis focuses on the evaluation in 2000 (*n* = 682 with complete data including carotid ultrasound), which served as the baseline for this analysis, and on the 10-yr follow-up period between 2000 and 2010. Follow-up for clinical endpoints was 100% complete (*n* = 682, mean and median follow up time 8.6 and 10 yr), whereas follow up ultrasound examinations in 2005 and 2010 were available in 558 men and women (i.e., 91.9% of survivors or 81.8% overall). The study protocol was approved by the Ethics Committees of Verona and Bolzano, and all participants gave their written informed consent before entering the study.

All risk factors were assessed by means of validated standard procedures described previously (Kiechl et al., 2002, 2013; Stegemann et al., 2014). In brief, body mass index was calculated as weight divided by height squared (kg/m^2^). Hypertension was defined as blood pressure ≥140/90 mm Hg (mean of three independent readings obtained with a standard mercury sphygmomanometer after at least 10 min of rest) or the use of antihypertensive drugs. Lifetime smoking was assessed as pack-years. Diabetes was defined based on American Diabetes Association criteria.

The composite CVD endpoint was composed of ischemic stroke, medical record–confirmed transient ischemic attack (TIA), myocardial infarction, and vascular death. A total of 102 individuals experienced primary outcome events. The extended composite endpoint additionally considered revascularization procedures, which increased the number of individuals affected to 118. Myocardial infarction was deemed confirmed when World Health Organization criteria for definite disease status were met. Ischemic stroke and TIA were classified according to the criteria of the National Survey of Stroke. A TIA was considered only if the diagnosis could be made with high accuracy (medical record–confirmed TIA). All revascularization procedures (angioplasty and surgery) were carefully recorded. Ascertainment of events or procedures did not rely on hospital discharge codes or the patient’s self-report but on a careful review of medical records provided by the general practitioners, death certificates, Bruneck Hospital files, and the extensive clinical and laboratory examinations performed as part of the study protocols. Major advantages of the Bruneck Study are that virtually all subjects living in the Bruneck area were referred to the local hospital and that the network existing between the local hospital and the general practitioners allowed retrieval of practically all medical information on persons living in the area. We also collected detailed information on the date, causes, and circumstances of death for all study subjects who did not survive the entire follow-up period by consulting death certificates, all medical records ever compiled on study subjects, and autopsy reports in the rare event of unexpected death. We were able to ascertain 100% of deaths and reliably classify them as vascular deaths, cancer deaths, or deaths from other causes (primary cause of death). Vascular mortality included deaths from ischemic stroke, myocardial infarction, rupture of aortic aneurysms, and sudden cardiac deaths. The experienced researcher who categorized all deaths and cardiovascular endpoints was unaware of laboratory data. There was no loss of follow-up for clinical endpoints.

#### Assessment of atherosclerosis

At each study visit, participants underwent bilateral carotid duplex sonography using a 10-MHz transducer and a 5-MHz Doppler. All subjects were examined in supine position. The scanning protocol involved four segments of the right and left carotid artery: proximal common carotid artery (15–30 mm proximal to the carotid bulb), distal common carotid artery (<15 mm proximal to the carotid bulb), proximal internal carotid artery (carotid bulb and the initial 10 mm of the vessel), and distal internal carotid artery (>10 mm above the flow divider). A plaque was defined as a focal structure encroaching into the arterial lumen with a thickening of the vessel wall of at least 0.5 mm relative to surrounding segments. The maximum axial diameter of plaques (in millimeters) was assessed on the near and far walls at each of the eight vessel segments. The atherosclerosis summation score was calculated by summing all diameters (intra-observer coefficient of variation, 13.5%; *n* = 100).

A person-based atherosclerosis progression model (Kiechl and Willeit, 1999a; Kiechl et al., 1999b) was developed and validated in the Bruneck Study that allowed differentiation between early and advanced stages in atherosclerosis development (Kiechl et al., 2013). Early atherosclerosis subsumes the manifestation of new plaques and/or nonstenotic progression of existing plaques. Main characteristics are a slow and continuous plaque growth, usually affecting more than one plaque simultaneously and accompanied by a compensatory or even overcompensatory enlargement of the vessel at the plaque site. The term incipient atherosclerosis was used for the development of first plaques in subjects free of atherosclerosis at baseline (Kiechl et al., 2002). Advanced complicated atherosclerosis was assumed when the relative increase in the maximum plaque diameter exceeded twice the measurement error for the method (internal carotid artery, 25%; common carotid artery, 17%) and a lumen narrowing of at least 40% was achieved. This process is featured by a usually solitary prominent increase in plaque size and absence of vascular remodeling, thus resulting in significant lumen compromise. From a mechanistic perspective, it refers to plaque destabilization and subsequent atherothrombosis (Willeit et al., 2000).

The two progression categories were highly reproducible (κ coefficients > 0.8 [*n* = 100]). Risk profiles differ significantly between the two stages of carotid artery disease with early atherosclerosis relying on standard risk factors and advanced atherosclerosis being mainly related to markers reflecting plaque vulnerability or enhanced prothrombotic activity (Willeit et al., 2000; Kiechl et al., 2007).

#### Statistical methodology

We tested the hypothesis that baseline ECP level is associated with new-onset CVD and advanced atherosclerosis (2000–2010), both of which rely on atherothrombosis, but not with early atherosclerosis driven by inflammation and standard vascular risk factors. Cox proportional hazard models with progressive adjustment were used to analyze time-to-event data on the primary CVD endpoint (ischemic strokes, medical record–confirmed TIAs, myocardial infarctions, and vascular deaths), extended CVD endpoint (plus revascularization procedures), and individual disease endpoints (stroke and TIA or myocardial infarction; Fig. 1 A). Subjects who suffered a CVD event were censored with respect to subsequent follow-up as were participant who died from nonvascular causes. We detected no departure from the proportional hazards assumption by inspecting Schoenfeld residuals and checking the parallelism of log–log survival plots. Associations between ECP and the various measures of atherosclerosis were tested by means of logistic regression analysis (Fig. 1 B) because the quinquennial examinations in the Bruneck Study provide reliable information on plaque development/progression in this time interval but no time-to-event information.

Base models were adjusted for age, sex, and either prior CVD (CVD endpoints) or baseline atherosclerosis (ultrasound endpoints). Multivariable analyses were additionally adjusted for hypertension, smoking (pack-years), diabetes, log_e_-transformed C-reactive protein, body-mass index, and LDL and high-density lipoprotein (HDL) cholesterol. We modeled ECP as a continuous variable and calculated hazard and odds ratios for a 1-SD unit–higher level of ECP.

In subsidiary analyses, we excluded subjects with prior CVD, eosinophil fractions >5%, or platelet inhibitor therapy. All p-values were two sided, and an α level of 0.05 was used. Analyses were conducted using SPSS and R 3.2.2 (R Foundation for Statistical Computing).

#### Proteomic serum analysis

Blood samples were drawn in the year 2000 after an overnight fast and 12 h of abstinence from smoking and immediately frozen and stored at −70°C (without any thawing-freezing cycle). Laboratory parameters were measured by standard assays, and a blood differential was performed using an automated analyzer (Kiechl et al., 2002, 2013; Stegemann et al., 2014). Eosinophil cationic protein (ECP) was measured in plasma samples collected in the year 2000 as part of a novel proteomics chip (Proximity Extension Assay; 4 Proseek Multiplex CVD I96 × 96; Olink Bioscience) as described previously (Assarsson et al., 2014).

### Animals

Animal experiments were approved by the government of Mittelfranken, the Mayo Clinic, and the Ludwig Maximilian University, Munich. 12/15-LO–deficient (*Alox15^−/−^*; C57BL/6 background), ΔdblGata1 (BALB/c background), and PHIL mice were described previously (Yu et al., 2002; Lee et al., 2004). ΔdblGata1mice carry a deletion of a high-affinity GATA-binding site in the GATA-1 promoter (Δ*dblGATA-1* mice), whereas PHIL mice express a diphtheria toxin A transgene that is driven by a fragment of the eosinophil peroxidase promoter. Both mouse strains show a selective and complete absence of the eosinophilic lineage. To achieve an eosinophil-specific deletion of *Alox15*, we crossed an EPX-Cre mouse (Doyle et al., 2013) with mice carrying floxed Alox15 alleles (both C57BL/6 background; Cole et al., 2012). Experiments were performed at an age of 8–10 wk. Whereas experiments with Δ*dblGATA-1* mice were performed in hemizygous mutant males and WT male littermates, the experiments with other mouse strains were performed with an equal gender distribution of mutant and WT mice. Animal experiments were performed by a blinded investigator.

### Cell culture

Eosinophils were generated from bone marrow isolated from 8-wk-old mice as previously described (Dyer et al., 2008) with minor modifications. In brief, bone marrow was incubated in RPMI medium (Gibco) containing 20% heat-inactivated feral calf serum (FCS), 25 mM Hepes, 100 IU/ml penicillin (Gibco), 10 µg/ml streptomycin (Gibco), 2 mM glutamine (Gibco), 1× nonessential amino acids (NEAA; Sigma-Aldrich), 1 mM sodium pyruvate (Sigma-Aldrich), 50 µM β-mercaptoethanol (Gibco), 100 ng/ml mFLT3L (PeproTech), and 100 ng/ml mouse stem cell factor (PeproTech) for 4 d, followed by 10-d differentiation with 10 ng/ml IL-5 (PeproTech). Half of the medium was changed every other day. Maturation was monitored by flow cytometry of SiglecF and CCR3 expression. Fully matured eosinophils were used after 14 d of total culture. For thrombin generation assays, RAW 264.7 mouse macrophages were treated with 100 ng/ml LPS in RPMI medium containing 10% FCS for 24 h.

### Plasma clotting time

Human standard citrate plasma (Siemens Healthcare) was freed from residual cellular and subcellular particles by ultracentrifugation and supplemented with 10% rat plasma (GeneTex). Plasma clotting time with or without addition of eosinophils was automatically recorded after recalcification with 20 µl of star-tem (ROTEM; Tem International GmbH) using a ball coagulometer (BC1; SYCOmed) at 37°C. When indicated, eosinophils were stimulated with 40 µM ADP (Roche) or 20 µg/ml collagen (Roche) for 10 min at 37°C before recalcification. Clotting index was calculated as follows: clotting time [sec.]^−1^ × 100.

### Calibrated thrombin and FXa generation

Thrombin generation was performed on a Fluoroskan Ascent FL Microplate Fluorometer and Luminometer (Thermo Fisher Scientific) by using a filter pair with excitation at 390 nm and emission at 460 nm. To each well of a 96-well plate containing 80 µl of eosinophils (250,000 cells) resuspended in plasma (10% rat/90% human), 20 µl of trigger solution (PBS, ADP, or collagen and/or phospholipids) was added and incubated for 10 min at 37°C. End concentration of ADP (Roche) was 40 µM. Then, 20 µl of recalcification solution containing a fluorogenic substrate for thrombin (Z-Gly-Gly-Arg) was automatically added. α_2_-Macroglobulin–thrombin was used separately for calibration according to the manufacturer’s specifications (Thermo Fisher Scientific). FXa generation was performed equally but with the fluorogenic substrate Pefafluor FXa (Loxo GMBH) and a filter pair with excitation at 340 nm and emission at 440 nm. When indicated, eosinophils were pretreated with 1 µM baicalein (EMD Millipore) or DMSO control and 40 µg/ml anti–mouse coagulation factor III/TF antibody (AF3178; R&D Systems) or isotype control (normal goat IgG; AB-108-C; R&D Systems) for 1 h at 37°C before stimulation. For thrombin generation with platelet-rich plasma (PRP) or platelet-poor plasma (PPP), PRP reagent (final concentration 0.5 pM TF; cat. no. TS42.00; Thrombinoscope) or PPP reagent (final concentration 5 pM TF and 4 mM phospholipids; cat. no. TS30.00; Thrombinoscope) was added according to the manufacturer’s specifications. Data were evaluated using Thrombinoscope 5.0 software.

### Calcium signaling

500,000 cells were stained with 6 µM Fluo-3 (Invitrogen) and 12 µM FuraRed (Invitrogen) in 200 µl of staining buffer (RPMI; 10 mM Hepes and 2% FCS) for 45 min at 37°C with gentle agitation. Cells were subsequently washed twice in Ca^2+^-free Tyrode’s buffer (134 mM NaCl, 12 mM NaHCO_3_, 2.9 mM KCl, 0.34 mM Na_2_HPO_4_, 1 mM MgCl_2_, 10 mM Hepes, 5 mM Hepes, and 0.5% BSA, pH 7.4) and incubated for 20 min at room temperature in the dark. Where indicated, CaCl_2_ was added to a final concentration of 1 mM. Calcium signaling experiments were performed at room temperature with a Gallios flow cytometer (Beckman Coulter) recording the Fluo-3/FuraRed ratio over time. Data were analyzed using FlowJo software (Tree Star). The reagents used were: BAPTA/AM and thapsigargin (EMD Millipore), tannic acid, and A23187 (Sigma-Aldrich).

### Antibodies

Rat anti–mouse SiglecF PE (E50-2440; BD), rabbit polyclonal anti–12-LO (ab23678; Abcam), rabbit anti–mouse TF (EPR8986; Abcam), polyclonal goat anti–mouse coagulation factor III/TF antibody (no. AF3178; R&D Systems), goat anti–rabbit Cy5 or Cy3 (Dianova), anti–mouse CD11b PE/Cy7 (M1/70), anti–human CD16 FITC (3G8), anti–mouse Ly6G FITC (1A8), anti–mouse CD41 PE or APC (MWReg30), anti–mouse CD115 PE or APC (AFS98), anti–mouse CD45 APC/Cy7 (30-F11), and anti–mouse CD16/32 (TruStain fcX) were used. Annexin V FITC and DAPI staining were purchased from BioLegend.

### FACS and flow cytometry

Bone marrow was isolated from sacrificed WT mice, and blood was drawn from the IVC of anaesthetized mice using a syringe (23-G needle) containing 10% 0.1 M sodium citrate. Red blood cells were gently lysed with ice-cold distilled water. Cells were pelleted and resuspended in buffer (1× PBS containing 0.5% BSA and 2 mM EDTA). After Fc receptor blockade, cells were stained with antibodies as indicated. For intracellular staining of 12/15-LO in blood leukocytes, cells were fixed and permeabilized before staining using a intracellular staining kit (00-5523; eBioscience). FACS was performed on a MoFlo XDP cell sorter (Beckman Coulter), and flow cytometry was performed on a Gallios flow cytometer (Beckman Coulter). Data were analyzed using FlowJo software. Platelet counts were recorded from citrated whole blood with an Adivia 120 cytometer (Siemens Healthcare).

### Immunofluorescence microscopy

For microscopy, thrombi were only partially dissected from the vessels to increase stability during preparation of the histological sections. Samples were embedded in Tissue-Tek optimal cutting temperature compound (Sakura), snap frozen, and stored at −80°C. 4-µm cryosections were fixated and permeabilized with ice-cold acetone and stored at −20°C until staining. Slides were stained with the indicated antibodies (dilution 1:100) and analyzed on an ECLIPSE Ni-U microscope (Nikon). Pictures were processed using NIS-Elements software (BR4.0; Nikon). For quantification of eosinophils in mouse thrombi, we analyzed thrombi of three independent experiments (four slides per individual thrombus).

### Isolation of platelets and generation of PRP and PPP

To avoid artificial platelet activation, blood was carefully drawn from the IVC of anaesthetized mice, using a syringe (23-G needle) containing 10% 0.1 M sodium citrate. To isolate platelets, a same volume of EDTA-Hepes-saline buffer (150 mM NaCl, 1 mM EDTA, and 10 mM Hepes) was added to the citrated blood, followed by two 20-min centrifugations at 50 *g*. Platelets were pelleted for 5 min at 300 *g* and resuspended in HBSA buffer (20 mM Hepes, 100 mM NaCl, and 1 mg/ml BSA) for further analysis. PRP was generated out of citrated plasma by 10-min centrifugation at 50 *g* and diluted to 140 cells/nl with autologous plasma. PPP was generated by two 10-min centrifugations at 1,500 *g*.

### Isolation of human eosinophils

Blood was drawn from healthy, nonatopic volunteers into syringes containing 10% 0.1 M citrate and was immediately processed. Cells were separated by Ficoll gradient centrifugation (Lymphoflot no. 824012; Bio-Rad Laboratories) according to the manufacturer’s specifications. From now on, all procedures described were performed at 4°C. The granulocyte-rich red pellet was subsequently lysed using ice-cold distilled water. Untouched eosinophils were isolated using a commercially available eosinophil isolation kit (130-092-010; Miltenyi Biotec) according to the manufacturer’s specifications. Purity of enriched eosinophils was evaluated by flow cytometry of side scatter and CD16 expression. Cells were resuspended in RPMI medium containing 10% FCS and stimulated with ADP or baicalein for 30 min at 37°C. Then, cells were washed and resuspended in HBSA buffer, and cell lysates were generated by three repeated freeze-thaw cycles. Samples were stored at −80°C until further analysis.

### Western blotting

Cells were washed twice with PBS and then lysed in radioimmunoprecipitation assay (RIPA) buffer (50 mM Tris, 150 mM NaCl, 1 mM EDTA, 1% Triton X-100, 1% sodium deoxycholate, and 0.1% SDS) containing 1% protease/phosphatase inhibitor (P9599; Sigma-Aldrich) and 1 mM PMSF (Active Motif) for 30 min on ice. Aortas were rinsed three times with PBS containing 1,000 IU heparin, cut into little pieces, and digested in serum-free DMEM containing 2 mg/ml collagenase type 2 (Worthington Biochemical Corporation) for 1 h at 37°C and then lysed in RIPA buffer for 30 min on ice. Protein content was assessed using a BCA Protein Assay kit (Thermo Fisher Scientific) according to the manufacturer’s specifications. Protein extracts were separated by SDS-PAGE using a 10% SDS-polyacrylamide gel, transferred to a Trans-Blot Nitrocellulose membrane (Bio-Rad Laboratories), and immunoblotted overnight in TBS-Tween containing 5% nonfat dry milk with the following antibodies: rabbit polyclonal anti–12-LO (ab23678; dilution 1:1,000; Abcam), mouse coagulation factor III/TF antibody (no. AF3178; dilution 1:200; R&D Systems), and rabbit anti–mouse β-actin (clone AC-74; dilution 1:1,000; Sigma-Aldrich). As a positive control for TF expression, lysates of LPS-treated RAW 264.7 mouse macrophages were used. As a positive control for 12/15-LO, mouse-resident macrophages were isolated from the peritoneal cavity of C57BL/6 mice by lavage, plated in RPMI medium, and washed thoroughly after 1 h to remove any nonadherent cells. After overnight culture in RPMI medium containing 10% FCS, cells were lysed in RIPA buffer and stored at −20°C until further analysis.

### Real-time PCR analysis

RNA was isolated with peqGOLD TRIFast (peqlab), and 500 ng of total RNA was reverse transcribed with human leukemia virus reverse transcription using the Gene Amp RNA PCR kit (Applied Biosystems) and oligo deoxythymidine primers. For quantitative RT-PCR after FACS, leukocyte subpopulations were directly sorted into lysis buffer, and RNA isolation was performed with an RNeasy Mini kit (QIAGEN) including DNA digestion (DNase I; no. EN0521; Fermentas) according to the manufacturer’s specifications. Quantitative real-time PCR was performed using a LightCycler instrument and SYBR Green I kit (Roche). We normalized the ratio of mRNA expression of the gene of interest to the mRNA expression for housekeeping genes *Actb* or *Gapdh* (with comparable results); depicted if not otherwise stated are the results for normalization to *Actb* (2^−▵Ct^). For detection of TF mRNA, two different primer pairs were used with comparable results; depicted in the paper are the results for primer pair 1. Used PCR primer sequences for mouse mRNA were: *Actb* forward, 5′-TGTCCACCTTCCAGCAGATGT-3′ and reverse, 5′-AGCTCAGTAACAGTCCGCCTAGA-3′; *Gapdh* forward, 5′-CTACACTGAGGACCAGGTTGTCT-3′ and reverse, 5′-CAGGAAATGAGCTTGACAAAGTT-3′; *Alox5* forward, 5′-ATTGCCATCCAGCTCAACCA-3′ and reverse, 5′-ACTGGAACGCACCCAGATTT-3′; *Alox12* forward, 5′-CGCTGTTGCCACCATGAGAT-3′ and reverse, 5′-ATGAGCTGGGTCCGCGTTC-3′; *Alox15* forward, 5′-CTCTCAAGGCCTGTTCAGGA-3′ and reverse, 5′-GTCCATTGTCCCCAGAACCT-3′; *Ccr3* forward, 5′-ACTGGACTCATAAAGGACTTAGCA-3′ and reverse, 5′-CCATGACCCCAGCTCTTTGA-3′; *Epx* forward, 5′-CGCCTGGATAGCCAGTATCG-3′ and reverse, 5′-ATGGAATCCTGCCGGTTCAG-3′; *Siglecf* forward, 5′-TCAGCCCTGAAAGTAGCAGC-3′ and reverse, 5′-TTTGGGTGTCTGGGACTGTG-3′; *F3* (coagulation factor III; TF) no. 1 forward, 5′-AGGATGTACCTGGGCCTAT-3′ and reverse, 5′-GGCTGTCCAAGGTTTGTGTC-3′; and *F3* (coagulation factor III; TF) no. 2 forward, 5′-GAAACTGGAAAAACAAGTGCTTCTT-3′ and reverse, 5′-CCAGGTCACATCCTTCACGAT-3′. Used PCR primer sequences for human mRNA were: *Alox15* forward, 5′-GTGTCCACTGGGGCCTCGCT-3′ and reverse, 5′-GCGGCCCCAGATACTCCGGTA-3′; *Alox5* forward, 5′-GACGTTCACGGCCGAGGTGG-3′ and reverse, 5′-AGCTGGCCGAAGTTGACCGC-3′; *Actb* forward, 5′-AGAAAATCTGGCACCACACC-3′ and reverse, 5′-TAGCACAGCCTGGATAGCAA-3′; and *Gapdh* forward, 5′-TGATGACATCAAGAAGGTGGTGAAG-3′ and reverse, 5′-TCCTTGGAGGCCATGTGGGCCAT-3′.

### Lipid extraction

10 ng 1,2-dimyristoyl-PE (DMPE) and 5 ng 15-HETE-d8 were added to each sample before extraction as an internal standard. Hydroperoxides were reduced to the corresponding alcohol by adding 1 mM SnCl_2_ for 10 min at room temperature. Lipids were extracted by adding 1 M acetic acid and 2-propanol hexane (2:20:30, vol/vol) to the sample at a ratio of 2.5 ml of solvent to 1 ml of sample by vortexing and then adding 2.5 ml of hexane. After vortexing and centrifugation at 1,500 rpm for 5 min, lipids were recovered in the upper hexane layer. Then, the samples were reextracted by the addition of an equal volume of hexane followed by further vortexing and centrifugation. The combined hexane layers were then dried under vacuum and analyzed for HETE-PEs and free eicosanoids using liquid chromatography tandem mass spectrometry (LC/MS/MS).

### HETE-PE quantitation using LC/MS/MS

Lipids were separated on a C18 Luna with a 3 µm or 150 mm × 2 mm column (Phenomenex), using a gradient of 50–100% B over 10 min followed by 30 min at 100% B (solvent A: methanol/acetonitrile/water, 1 mM ammonium acetate, 60:20:20; solvent B: methanol, 1 mM ammonium acetate) with a flow rate of 200 µl/min. Lipids were monitored using multiple reaction–monitoring mode. Transitions monitored were for parent ions of m/z 738.6, 764.6, 766.6, and 782.6 [M−H]− fragmenting to daughter ions with m/z 219.2 (15-HETE), 115.1 (5-HETE), or 179.1 (12-HETE). Standard curves were generated using internal standards (DMPE) and different synthetic primary standards. Products were quantified by LC/MS/MS electrospray ionization on an Applied Biosystems 4000 Q-Trap system. Acquisition of product ion spectra was triggered during elution of ions of interest, with the instrument operating in ion trap mode.

### Free eicosanoid quantitation using LC/MS/MS

Lipids were separated on a C18 Spherisorb ODS2 column (150 × 4.6 mm; 5 µm particle; Waters Ltd) using a gradient of 50–90% B over 30 min, followed by 5 min at 90% B (A, water/acetonitrile/acetic acid, 75:25:0.1; B, methanol/acetonitrile/acetic acid, 60:40:0.1) with a flow rate of 1 ml/min. Eicosanoid species were monitored with specific parent to daughter ion transitions in negative ion mode ([M−H]−) for HETEs (m/z 319.2) at 115 (5-HETE), 179.1 (12-HETE), 219 (15-HETE), 155 (8-HETE), and 167 (11-HETE). 15-HETE-d8 was monitored at m/z 327 to 226. Products were identified and quantified using primary standards, and internal standard runs in parallel under the same method conditions.

### Quantification of externalized aminophospholipids

Externalization of PE and PS species in eosinophils was measured according as previously described (Thomas et al., 2014). In brief, cultured mouse eosinophils (4 × 10^6^ per ml) were stimulated with ADP (40 µM) or the calcium ionophore A23187 (10 µM) and treated with EZ-link NHS-biotin or EZ-link sulfo-NHS-biotin (Thermo Fisher Scientific) for measuring total cellular lipids and external aminophospholipids, respectively, by LC/MS/MS.

### Generation of liposomes

Liposomes used in the thrombin generation experiments contained 20% of the indicated oxidized or unoxidized phospholipid species and 75% PAPC and 5% PAPS as carrier lipids. Phospholipids were solved in methanol or chloroform and kept under a layer of argon on −80°C until usage.

Phospholipids used were: PAPS (110670; Avanti Polar Lipids, Inc.), 1-hexadecanoyl-2-(5Z,8Z,11Z,14Z-eicosatetraenoyl)-*sn*-glycero-3-phospho-l-serine PAPE (110638; Avanti Polar Lipids, Inc.), 1-hexadecanoyl-2-(5Z,8Z,11Z,14Z-eicosatetraenoyl)-*sn*-glycero-3-phosphoethanolamine PAPC (850459C; Avanti Polar Lipids, Inc.), and 1-hexadecanoyl-2-(5Z,8Z,11Z,14Z-eicosatetraenoyl)-*sn*-glycero-3-phosphocholine 12-HETE-PE. Lipids were generated as previously described (Morgan et al., 2010).

Indicated phospholipids were added to PAPC/PAPS carrier lipids before solvent was evaporated under a gentle stream of Argon. Phospholipids were resuspended in HBSA buffer and generously vortexed. Then, liposomes were prepared by 10 freeze-thaw cycles and kept on ice before performing the assay.

### Tail-bleeding assays

Two different tail-bleeding assays were performed as previously described (Liu et al., 2012; Rossaint et al., 2013) with minor modifications. In brief, mice were anaesthetized with ketamine (100 mg/kg body weight) and xylazine (20 mg/kg body weight) and placed under a heating lamp to maintain a constant body temperature of 37°C. A 3-mm (ca. 1–1.2-mm diameter) or 15-mm (ca. 2.2–2.5-mm diameter) piece of the tail tip was cut off with a sharp scalpel. The distal 2.5 cm of the bleeding tail was immediately dipped into a 50-ml tube filled with prewarmed 0.9% saline, and the time until cessation of blood flow for >5 s was recorded. If not otherwise stated, bleeding was monitored for 20 min in total to assess rebleedings caused by lacking thrombus stability. The amount of lost blood was assessed by comparison of body weight (including tail tip) before and after bleeding (relative weight loss). Additionally, the collected blood was lysed, and OD_575nm_ was recorded, indicating the hemoglobin content of the lost blood.

### Injury-related venous thrombosis

Thrombosis was induced as described previously (Wang et al., 2006) with minor modifications. In brief, mice (20–25 g body weight) were anaesthetized with ketamine (100 mg/kg body weight) and xylazine (20 mg/kg body weight) and placed under a heating lamp to maintain a constant body temperature of 37°C. A ventral midline incision was performed, and the intestines were gently put aside. The IVC was laid free carefully, and a filter paper (1 × 2 × 4 mm) soaked with 4% aqueous ferric chloride solution was placed on top of the vessel. After 3-min incubation, the filter paper was removed, and the peritoneal cavity was thoroughly rinsed with prewarmed 0.9% saline. After another 30 min, mice were sacrificed, blood was taken by cardiac puncture, and the vena cava containing the thrombus was removed. The clot was dissected free from the vessel and prepared under a microscope for further analysis. Wet thrombus weight was measured using a precision balance (Sartorius R16P) after removal of excess water.

### Injury-related arterial thrombosis and intravital microscopy

Mouse platelets were isolated from whole blood, labeled with 5-carboxy-flourescein diacetate succinimidyl ester, and adjusted to a final concentration of 150 × 106 platelets/250 µl. Mice were anesthetized (Medetomidin, 0.5 mg/kg body weight; Midazolam, 5 mg/kg body weight; and Fentanyl, 0.05 mg/kg body weight), and the platelet suspension was injected i.v. via a jugular vein catheter. The contralateral carotid artery was prepared under a dissecting microscope (Stemi 2000-CS; ZEISS), and a filter paper (1 × 2 × 4 mm) presoaked in 10% FeCl_3_ solution was placed on top of the vessel. After 3-min incubation, the filter paper was removed, and dynamic thrombus formation was recorded over time using a high-speed wide-field fluorescence microscope (BX51WI; Olympus) equipped with a long-distance condenser and a 20× (NA 0.95) water immersion objective, a monochromator (MT 20; Olympus), and a charge-coupled device camera (ORCA-ER; Hamamatsu Photonics). For image acquisition and analysis, a computer (Dell) with Cell^R software (Olympus) was used.

### IVC flow restriction model

The IVC flow restriction model was previously described in detail (von Brühl et al., 2012). In brief, a median laparotomy was performed, and the IVC was exposed by atraumatic surgery. We positioned a space holder (FloppyR II Guide Wire 0.014 in [0.36 mm]; Guidant Corporation) on the outside of the vessel and placed a permanent narrowing ligature (8.0 monofil polypropylene filament, Premilene; Braun) exactly below the left renal vein. Subsequently, the wire was removed to avoid complete vessel occlusion. Side branches were not ligated or manipulated. Flow velocity was determined immediately after the flow restriction (Cap-Image 7.1). Because we wanted to rule out endothelial injury as a trigger for venous thrombosis, all mice with bleedings or any injury of the IVC during surgery were excluded from further analysis. There was no difference in the exclusion rate across the different experimental groups. The median laparotomy was immediately sutured by a 7.0 polypropylene suture (Ethicon). For weight measurement, the vessel was excised just below the renal veins and proximal to the confluence of the common iliac veins. After the restriction procedure, the blood flow velocity was reduced by ∼80% (Fig. 1 B). The shear stress was 0.144 dyne/cm^2^ ± 0.02 SEM before the flow restriction and 0.072 dyne/cm^2^ ± 0.017 SEM after the procedure in the IVC close to the site of ligation. Sham experiments consisted of preparation of the IVC and placement of the filament under the vessel without ligation.

### Depletion of platelets and eosinophils and baicalein treatment

For depletion of eosinophils, 20 µg of purified rat anti–mouse SiglecF (E50-2440; BD) or isotype rat IgG2a (BD) in 100 µl of sterile PBS was injected into the lateral tail vein. Experiments were performed 6 h after injection. For depletion of platelets, anti–platelet antibody (6A6-IgG2a; 0.2 µg/g body weight) or isotope IgG2a was injected into the lateral tail vein (both antibodies were provided by F. Nimmerjahn, Friedrich-Alexander-University Erlangen-Nürnberg, Erlangen, Germany). Experiments were performed 1 h after injection. Depletion of platelets and eosinophils was evaluated by an Advia hemocytometer or Gallios flow cytometer (Beckman Coulter), respectively, in separate experiments after the indicated time to avoid any interference with the in vivo assays by the vascular injury. For inhibition of 12/15-LO in vivo, 10 µg/g body weight baicalein or DMSO control in 200 µl PBS was injected intraperitoneal two times within 24 h. Experiments were performed 1 h after last injection.

### ELISAs

Mouse blood was drawn into syringes containing 10% 0.1 M citrate and immediately put on ice. Plasma was obtained by 10-min centrifugation (3,000 *g*) and stored at −80°C until analysis. The following ELISA kits were used: Mouse TAT Complex ELISA kit (EMT1020-1; Assaypro) and Human ECP ELISA kit (SK00128-01; Aviscera Bioscience). ELISAs were performed according to the manufacturer’s specifications.

### Statistics

Data are shown as means ± SEM. Group mean values from in vivo experiments were compared by unpaired, two-tailed Student’s *t* test, and in vitro experiments were analyzed using one-way ANOVA (Bonferroni correction for multiple comparison). If not otherwise indicated, the data shown are representative of at least three experiments producing similar results. *, P < 0.05; **, P < 0.01; ***, P < 0.001.

### Online supplemental material

Table S1 shows a summary of the variables of the study cohort and includes additional data on the distribution of eosinophils within thrombi. Fig. S1 shows immunofluorescence imaging data of mouse thrombi. Fig. S2 includes data on the parameters of platelet function and plasmatic coagulation in Δ*dblGATA1* mice. Fig. S3 includes data on the used gating strategies, 12/15-LO expression in the vascular wall, and the procoagulatory potential of eosinophils. Fig. S4 shows additional data about parameters of platelet function and plasmatic coagulation in *Alox15^−/−^* mice.

## Supplementary Material

Supplemental Materials (PDF)

Video 1

Video 2

Video 3

Video 4

## References

[bib1] Ames P.R., Margaglione M., Mackie S., and Alves J.D. 2010 Eosinophilia and thrombophilia in churg strauss syndrome: a clinical and pathogenetic overview. Clin. Appl. Thromb. Hemost. 16:628–636. 10.1177/1076029609348647 19833618

[bib2] Assarsson E., Lundberg M., Holmquist G., Björkesten J., Thorsen S.B., Ekman D., Eriksson A., Rennel Dickens E., Ohlsson S., Edfeldt G., 2014 Homogenous 96-plex PEA immunoassay exhibiting high sensitivity, specificity, and excellent scalability. PLoS One. 9:e95192 10.1371/journal.pone.0095192 24755770 PMC3995906

[bib3] Bergmeier W., Weidinger C., Zee I., and Feske S. 2013 Emerging roles of store-operated Ca^2+^ entry through STIM and ORAI proteins in immunity, hemostasis and cancer. Channels (Austin). 7:379–391. 10.4161/chan.24302 23511024 PMC3913761

[bib4] Bouchard B.A., Mann K.G., and Butenas S. 2010 No evidence for tissue factor on platelets. Blood. 116:854–855. 10.1182/blood-2010-05-285627 20688968 PMC2918337

[bib5] Clark S.R., Thomas C.P., Hammond V.J., Aldrovandi M., Wilkinson G.W., Hart K.W., Murphy R.C., Collins P.W., and O’Donnell V.B. 2013 Characterization of platelet aminophospholipid externalization reveals fatty acids as molecular determinants that regulate coagulation. Proc. Natl. Acad. Sci. USA. 110:5875–5880. 10.1073/pnas.1222419110 23530199 PMC3625294

[bib6] Cole B.K., Morris M.A., Grzesik W.J., Leone K.A., and Nadler J.L. 2012 Adipose tissue-specific deletion of 12/15-lipoxygenase protects mice from the consequences of a high-fat diet. Mediators Inflamm. 2012:851798 10.1155/2012/851798 23326022 PMC3543811

[bib7] Cyrus T., Praticò D., Zhao L., Witztum J.L., Rader D.J., Rokach J., FitzGerald G.A., and Funk C.D. 2001 Absence of 12/15-lipoxygenase expression decreases lipid peroxidation and atherogenesis in apolipoprotein e-deficient mice. Circulation. 103:2277–2282. 10.1161/01.CIR.103.18.2277 11342477

[bib8] Doyle A.D., Jacobsen E.A., Ochkur S.I., Willetts L., Shim K., Neely J., Kloeber J., Lesuer W.E., Pero R.S., Lacy P., 2013 Homologous recombination into the eosinophil peroxidase locus generates a strain of mice expressing Cre recombinase exclusively in eosinophils. J. Leukoc. Biol. 94:17–24. 10.1189/jlb.0213089 23630390 PMC3685019

[bib9] Dyer K.D., Moser J.M., Czapiga M., Siegel S.J., Percopo C.M., and Rosenberg H.F. 2008 Functionally competent eosinophils differentiated ex vivo in high purity from normal mouse bone marrow. J. Immunol. 181:4004–4009. 10.4049/jimmunol.181.6.4004 18768855 PMC2680436

[bib10] Engelmann B., and Massberg S. 2013 Thrombosis as an intravascular effector of innate immunity. Nat. Rev. Immunol. 13:34–45. 10.1038/nri3345 23222502

[bib11] Flaumenhaft R. 2014 Thrombus formation reimagined. Blood. 124:1697–1698. 10.1182/blood-2014-06-579656 25214193 PMC4162099

[bib12] Heredia J.E., Mukundan L., Chen F.M., Mueller A.A., Deo R.C., Locksley R.M., Rando T.A., and Chawla A. 2013 Type 2 innate signals stimulate fibro/adipogenic progenitors to facilitate muscle regeneration. Cell. 153:376–388. 10.1016/j.cell.2013.02.053 23582327 PMC3663598

[bib13] Jacobsen E.A., Helmers R.A., Lee J.J., and Lee N.A. 2012 The expanding role(s) of eosinophils in health and disease. Blood. 120:3882–3890. 10.1182/blood-2012-06-330845 22936660 PMC3496950

[bib14] Kiechl S., and Willeit J. 1999 a The natural course of atherosclerosis. Part I: incidence and progression. Arterioscler. Thromb. Vasc. Biol. 19:1484–1490. 10.1161/01.ATV.19.6.1484 10364079

[bib15] Kiechl S., and Willeit J.. Bruneck Study Group 1999 b The natural course of atherosclerosis. Part II: vascular remodeling. Arterioscler. Thromb. Vasc. Biol. 19:1491–1498. 10.1161/01.ATV.19.6.1491 10364080

[bib16] Kiechl S., Lorenz E., Reindl M., Wiedermann C.J., Oberhollenzer F., Bonora E., Willeit J., and Schwartz D.A. 2002 Toll-like receptor 4 polymorphisms and atherogenesis. N. Engl. J. Med. 347:185–192. 10.1056/NEJMoa012673 12124407

[bib17] Kiechl S., Schett G., Schwaiger J., Seppi K., Eder P., Egger G., Santer P., Mayr A., Xu Q., and Willeit J. 2007 Soluble receptor activator of nuclear factor-κB ligand and risk for cardiovascular disease. Circulation. 116:385–391. 10.1161/CIRCULATIONAHA.106.686774 17620507

[bib18] Kiechl S., Wittmann J., Giaccari A., Knoflach M., Willeit P., Bozec A., Moschen A.R., Muscogiuri G., Sorice G.P., Kireva T., 2013 Blockade of receptor activator of nuclear factor-κB (RANKL) signaling improves hepatic insulin resistance and prevents development of diabetes mellitus. Nat. Med. 19:358–363. 10.1038/nm.3084 23396210

[bib19] Kuhn H., Banthiya S., and van Leyen K. 2015 Mammalian lipoxygenases and their biological relevance. Biochim. Biophys. Acta. 1851:308–330. 10.1016/j.bbalip.2014.10.002 25316652 PMC4370320

[bib20] Kühn H., and O’Donnell V.B. 2006 Inflammation and immune regulation by 12/15-lipoxygenases. Prog. Lipid Res. 45:334–356. 10.1016/j.plipres.2006.02.003 16678271

[bib21] Kunzelmann K., Nilius B., Owsianik G., Schreiber R., Ousingsawat J., Sirianant L., Wanitchakool P., Bevers E.M., and Heemskerk J.W. 2014 Molecular functions of anoctamin 6 (TMEM16F): a chloride channel, cation channel, or phospholipid scramblase? Pflugers Arch. 466:407–414. 10.1007/s00424-013-1305-1 23748496

[bib22] Lee J.J., Dimina D., Macias M.P., Ochkur S.I., McGarry M.P., O’Neill K.R., Protheroe C., Pero R., Nguyen T., Cormier S.A., 2004 Defining a link with asthma in mice congenitally deficient in eosinophils. Science. 305:1773–1776. 10.1126/science.1099472 15375267

[bib23] Lee J.J., Jacobsen E.A., McGarry M.P., Schleimer R.P., and Lee N.A. 2010 Eosinophils in health and disease: the LIAR hypothesis. Clin. Exp. Allergy. 40:563–575. 10.1111/j.1365-2222.2010.03484.x 20447076 PMC2951476

[bib24] Liu Y., Jennings N.L., Dart A.M., and Du X.J. 2012 Standardizing a simpler, more sensitive and accurate tail bleeding assay in mice. World J. Exp. Med. 2:30–36. 10.5493/wjem.v2.i2.30 24520531 PMC3905578

[bib25] Mackman N. 2004 Role of tissue factor in hemostasis, thrombosis, and vascular development. Arterioscler. Thromb. Vasc. Biol. 24:1015–1022. 10.1161/01.ATV.0000130465.23430.74 15117736

[bib26] McCarty O.J., Tien N., Bochner B.S., and Konstantopoulos K. 2003 Exogenous eosinophil activation converts PSGL-1-dependent binding to CD18-dependent stable adhesion to platelets in shear flow. Am. J. Physiol. Cell Physiol. 284:C1223–C1234. 10.1152/ajpcell.00403.2002 12529243

[bib27] Moosbauer C., Morgenstern E., Cuvelier S.L., Manukyan D., Bidzhekov K., Albrecht S., Lohse P., Patel K.D., and Engelmann B. 2007 Eosinophils are a major intravascular location for tissue factor storage and exposure. Blood. 109:995–1002. 10.1182/blood-2006-02-004945 17003379

[bib28] Morgan A.H., Hammond V.J., Morgan L., Thomas C.P., Tallman K.A., Garcia-Diaz Y.R., McGuigan C., Serpi M., Porter N.A., Murphy R.C., and O’Donnell V.B. 2010 Quantitative assays for esterified oxylipins generated by immune cells. Nat. Protoc. 5:1919–1931. 10.1038/nprot.2010.162 21127486 PMC3678246

[bib29] Nemerson Y. 1968 The phospholipid requirement of tissue factor in blood coagulation. J. Clin. Invest. 47:72–80. 10.1172/JCI105716 16695947 PMC297149

[bib30] O’Donnell V.B., Murphy R.C., and Watson S.P. 2014 Platelet lipidomics: modern day perspective on lipid discovery and characterization in platelets. Circ. Res. 114:1185–1203. 10.1161/CIRCRESAHA.114.301597 24677238 PMC4021279

[bib31] Riegger J., Byrne R.A., Joner M., Chandraratne S., Gershlick A.H., Ten Berg J.M., Adriaenssens T., Guagliumi G., Godschalk T.C., Neumann F.J., Prevention of Late Stent Thrombosis by an Interdisciplinary Global European Effort (PRESTIGE) Investigators 2016 Histopathological evaluation of thrombus in patients presenting with stent thrombosis. A multicenter European study: a report of the prevention of late stent thrombosis by an interdisciplinary global European effort consortium. Eur. Heart J. 37:1538–1549. 10.1093/eurheartj/ehv419 26761950 PMC4872283

[bib32] Rossaint J., Vestweber D., and Zarbock A. 2013 GDF-15 prevents platelet integrin activation and thrombus formation. J. Thromb. Haemost. 11:335–344. 10.1111/jth.12100 23231375

[bib33] Rothenberg M.E., and Hogan S.P. 2006 The eosinophil. Annu. Rev. Immunol. 24:147–174. 10.1146/annurev.immunol.24.021605.090720 16551246

[bib34] Stegemann C., Pechlaner R., Willeit P., Langley S.R., Mangino M., Mayr U., Menni C., Moayyeri A., Santer P., Rungger G., 2014 Lipidomics profiling and risk of cardiovascular disease in the prospective population-based Bruneck study. Circulation. 129:1821–1831. 10.1161/CIRCULATIONAHA.113.002500 24622385

[bib35] Stevens G., Mascarenhas M., and Mathers C. 2009 Global health risks: progress and challenges. Bull. World Health Organ. 87:646 10.2471/BLT.09.070565 19784438 PMC2739926

[bib36] Suzuki J., Umeda M., Sims P.J., and Nagata S. 2010 Calcium-dependent phospholipid scrambling by TMEM16F. Nature. 468:834–838. 10.1038/nature09583 21107324

[bib37] Tani Y., Isobe Y., Imoto Y., Segi-Nishida E., Sugimoto Y., Arai H., and Arita M. 2014 Eosinophils control the resolution of inflammation and draining lymph node hypertrophy through the proresolving mediators and CXCL13 pathway in mice. FASEB J. 28:4036–4043. 10.1096/fj.14-251132 24891522 PMC5395732

[bib38] Thomas C.P., Morgan L.T., Maskrey B.H., Murphy R.C., Kühn H., Hazen S.L., Goodall A.H., Hamali H.A., Collins P.W., and O’Donnell V.B. 2010 Phospholipid-esterified eicosanoids are generated in agonist-activated human platelets and enhance tissue factor-dependent thrombin generation. J. Biol. Chem. 285:6891–6903. 10.1074/jbc.M109.078428 20061396 PMC2844139

[bib39] Thomas C.P., Clark S.R., Hammond V.J., Aldrovandi M., Collins P.W., and O’Donnell V.B. 2014 Identification and quantification of aminophospholipid molecular species on the surface of apoptotic and activated cells. Nat. Protoc. 9:51–63. 10.1038/nprot.2013.163 24336470

[bib40] Tian Y., Schreiber R., and Kunzelmann K. 2012 Anoctamins are a family of Ca2^+^-activated Cl^−^ channels. J. Cell Sci. 125:4991–4998. 10.1242/jcs.109553 22946059

[bib41] Todd S., Hemmaway C., and Nagy Z. 2014 Catastrophic thrombosis in idiopathic hypereosinophilic syndrome. Br. J. Haematol. 165:425 10.1111/bjh.12729 24456103

[bib42] Uderhardt S., and Krönke G. 2012 12/15-lipoxygenase during the regulation of inflammation, immunity, and self-tolerance. J. Mol. Med. (Berl.). 90:1247–1256. 10.1007/s00109-012-0954-4 22983484

[bib43] von Brühl M.L., Stark K., Steinhart A., Chandraratne S., Konrad I., Lorenz M., Khandoga A., Tirniceriu A., Coletti R., Köllnberger M., 2012 Monocytes, neutrophils, and platelets cooperate to initiate and propagate venous thrombosis in mice in vivo. J. Exp. Med. 209:819–835. 10.1084/jem.20112322 22451716 PMC3328366

[bib44] Wang X., Smith P.L., Hsu M.Y., Ogletree M.L., and Schumacher W.A. 2006 Murine model of ferric chloride-induced vena cava thrombosis: evidence for effect of potato carboxypeptidase inhibitor. J. Thromb. Haemost. 4:403–410. 10.1111/j.1538-7836.2006.01703.x 16420573

[bib45] Willeit J., Kiechl S., Oberhollenzer F., Rungger G., Egger G., Bonora E., Mitterer M., and Muggeo M. 2000 Distinct risk profiles of early and advanced atherosclerosis: prospective results from the Bruneck Study. Arterioscler. Thromb. Vasc. Biol. 20:529–537. 10.1161/01.ATV.20.2.529 10669653

[bib46] Wolberg A.S. 2007 Thrombin generation and fibrin clot structure. Blood Rev. 21:131–142. 10.1016/j.blre.2006.11.001 17208341

[bib47] Yang H., Kim A., David T., Palmer D., Jin T., Tien J., Huang F., Cheng T., Coughlin S.R., Jan Y.N., and Jan L.Y. 2012 TMEM16F forms a Ca^2+^-activated cation channel required for lipid scrambling in platelets during blood coagulation. Cell. 151:111–122. 10.1016/j.cell.2012.07.036 23021219 PMC3582364

[bib48] Yu C., Cantor A.B., Yang H., Browne C., Wells R.A., Fujiwara Y., and Orkin S.H. 2002 Targeted deletion of a high-affinity GATA-binding site in the GATA-1 promoter leads to selective loss of the eosinophil lineage in vivo. J. Exp. Med. 195:1387–1395. 10.1084/jem.20020656 12045237 PMC2193547

