# Correction: Enzymatic lipid oxidation by eosinophils propagates coagulation, hemostasis, and thrombotic disease

**DOI:** 10.1084/jem.2016107002142018c

**Published:** 2018-03-05

**Authors:** Stefan Uderhardt, Jochen A. Ackermann, Tobias Fillep, Victoria J. Hammond, Johann Willeit, Peter Santer, Manuel Mayr, Markus Biburger, Meike Miller, Katie R. Zellner, Konstantin Stark, Alexander Zarbock, Jan Rossaint, Irene Schubert, Dirk Mielenz, Barbara Dietel, Dorette Raaz-Schrauder, Cihan Ay, Thomas Gremmel, Johannes Thaler, Christian Heim, Martin Herrmann, Peter W. Collins, Gernot Schabbauer, Nigel Mackman, David Voehringer, Jerry L. Nadler, James J. Lee, Steffen Massberg, Manfred Rauh, Stefan Kiechl, Georg Schett, Valerie B. O’Donnell, Gerhard Krönke

Vol. 214, No. 7, July 2017. https://doi.org/10.1084/jem.20161070

The authors regret that an error appeared in their original version of Fig. 4. Some of the PE and PS species listed on the x axis of Fig. 4 B were incorrect. The corrected figure and corresponding legend appear below.

**Figure 4.**
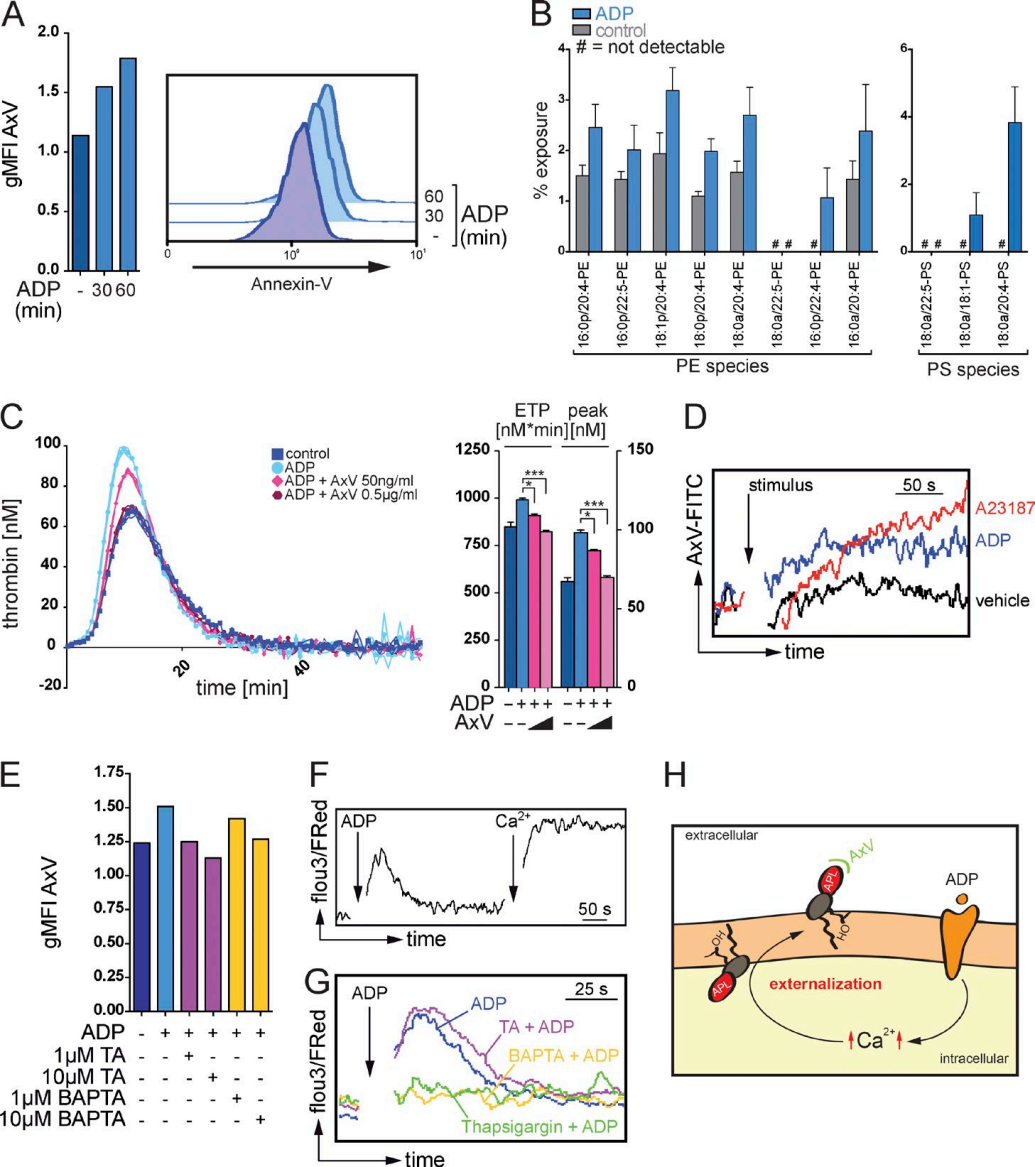
**Ca^2+^-dependent exposure of aminophospholipids by eosinophils promote thrombin generation.** (A) Flow cytometry analysis of the binding of annexin V (AxV) to aminophospholipids on the surface of resting or ADP-stimulated mouse eosinophils. Histograms show representative annexin V stainings, and bar graphs show mean geometric fluorescence intensities (gMFI). (B) LC/MS/MS-based quantification of the exposure of the aminophospholipids PE and PS in mouse eosinophils in response to ADP stimulation. (C, left) Calibrated thrombin generation assays with resting or ADP-stimulated mouse eosinophils in the presence of annexin V. (Right) Bar graphs show endogenous thrombin potential (ETP; nM*min) and peak of thrombin generation (peak; nM). (D) Flow cytometry analysis of annexin V binding on mouse eosinophils over time in the presence of calcium ionophore A23187, ADP, or vehicle. (E) Flow cytometry analysis of annexin V binding on mouse eosinophils in the presence of tannic acid (TA) or intracellular Ca^2+^-chelator BAP​TA/AM. Bar graphs show geometric mean fluorescence intensity. (F) Flow cytometry–based analysis of intracellular Ca^2+^ signaling, indicated by Fluo3/FuraRed ratio, over time in a Ca^2+^-free environment. Where indicated (arrow and Ca^2+^), CaCl_2_ at a final concentration of 1 mM was added. (G) Flow cytometry–based analysis of intracellular Ca^2+^ signaling, indicated by Fluo3/FuraRed ratio, over time in a Ca^2+^-free environment. (H) Postulated mechanism of a sequential generation and Ca^2+^-dependent externalization of aminophospholipids (APL) at the surface of eosinophils. OH indicates hydroxyl group. Data are representative of at least three independent experiments. Error bars represent SEM. *, P < 0.05; ***, P < 0.001.

The online HTML and PDF versions of this paper have been corrected. The error remains only in the print version.

