# Supplementary material for: Enzymatic lipid oxidation by eosinophils propagates coagulation, hemostasis, and thrombotic disease
Source: J Exp Med. 2017 Jul 3;214(7):2121–38. doi: 10.1084/jem.20161070 (PMC5502424; doi:10.1084/jem.20161070)
Supplement: Supplemental Materials (PDF) [file JEM_20161070_sm.pdf]

The Journal of Experimental Medicine

SUPPLEMENTAL MATERIAL

Uderhardt et al., https://doi.org/10.1084/jem.20161070

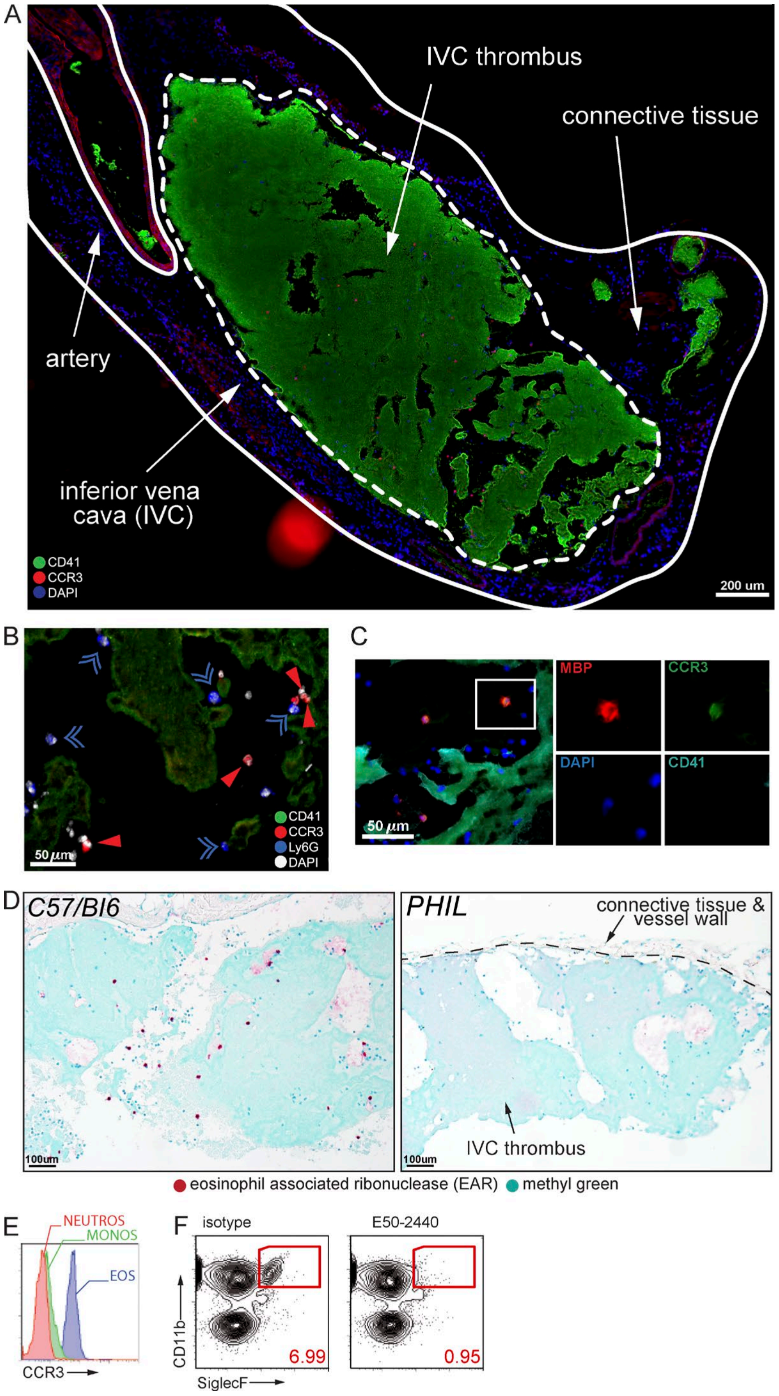

Figure S1. **Ex vivo imaging of eosinophil accumulation in experimentally induced mouse thrombi.** (A) Representative microscopy picture ($n = 5$) showing an annotated, longitudinal section of an isolated ferric chloride–induced thrombus of a mouse IVC with immunofluorescence staining for CD41 (green), CCR3 (red), and DAPI (blue). Bar, 200 µm. (B) Representative detail of a venous mouse thrombus stained for CD41 (green), CCR3 (red), Ly6G (blue), and DAPI (white). Red arrowheads indicate $CCR3^+$ eosinophils, and blue arrowheads indicate $Ly6G^+$ neutrophils. Bar, 50 µm. (C) Representative detail of a venous mouse thrombus stained for CCR3 (green), major basic protein (MBP; red), DAPI (blue), and CD41 (turquoise). The box indicates an MBP^+CCR3^+ eosinophil. Bar, 50 µm. (D) Representative microscopy picture ($n = 5$) showing a longitudinal section of an isolated ferric chloride–induced thrombus of a mouse IVC in WT and eosinophil-deficient *PHIL* mice ($n = 5$) stained for eosinophil-associated ribonuclease (EAR; red). (E) Histogram of flow cytometry analysis of FACS-sorted cells showing CCR3 expression in monocytes (MONOS), neutrophils (NEUTROS), or eosinophils (EOS) in the peripheral blood of WT mice. (F) Flow cytometry analysis of peripheral mouse eosinophils ($CD11b^+SiglecF^+$) 6 h after intravenous injection of isotope control (left) or anti-SiglecF antibody (E50-2440; right).

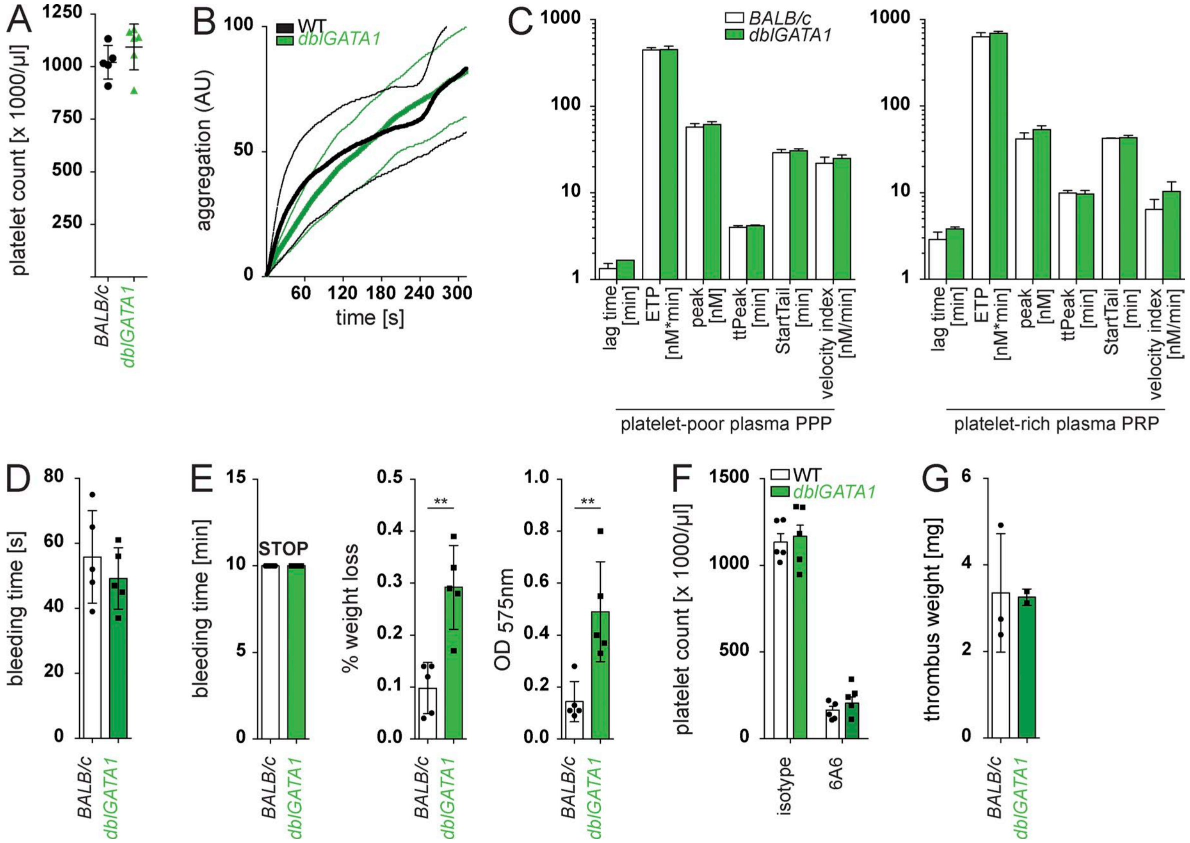

Figure S2. **Assessment of platelet function and coagulation capacity in eosinophil-deficient mice.** (A) Platelet counts in peripheral blood from WT (n = 5) or Δ*dblGATA1* mice (n = 6). (B) Platelet aggregation assay with whole blood from WT (n = 4) or Δ*dblGATA1* mice (n = 4). Thin lines represent SD. AU, arbitrary units. (C) Parameters obtained from coagulometric assays with PPP (left) or PRP (right) plasma isolated from BALB/c WT (white; n = 4) or ΔdblGATA1 (green; n = 4) mice. ETP, endogenous thrombin potential; ttPeak, time-to-peak. (D) 3-mm tail cut bleeding assays with WT (*BALB/c*; n = 5) and Δ*dblGATA1* (n = 9) mice. The bar graph shows primary bleeding time (time until the first stop of bleeding). (E) 3-mm tail cut bleeding assay with WT (n = 5) or Δ*dblGATA1* (n = 5) mice after platelet depletion by intravenous injection of 6A6 antibody. Bleeding experiments were terminated prematurely after 10 min. Graphs show bleeding time, relative weight loss, and OD_{575nm} of the collected blood after lysis. (F) Platelet counts in peripheral blood from BALB/c (white; n = 5) or Δ*dblGATA1* (green; n = 5) mice after injection of isotype control or 6A6. (G) Thrombus weight in WT (n = 3) or Δ*dblGATA1* (n = 3) mice upon stasis-induced thrombosis in the IVC flow restriction model. Data are representative of at least three independent experiments. Error bars represent SEM. **, $P < 0.01$; Student's t test.

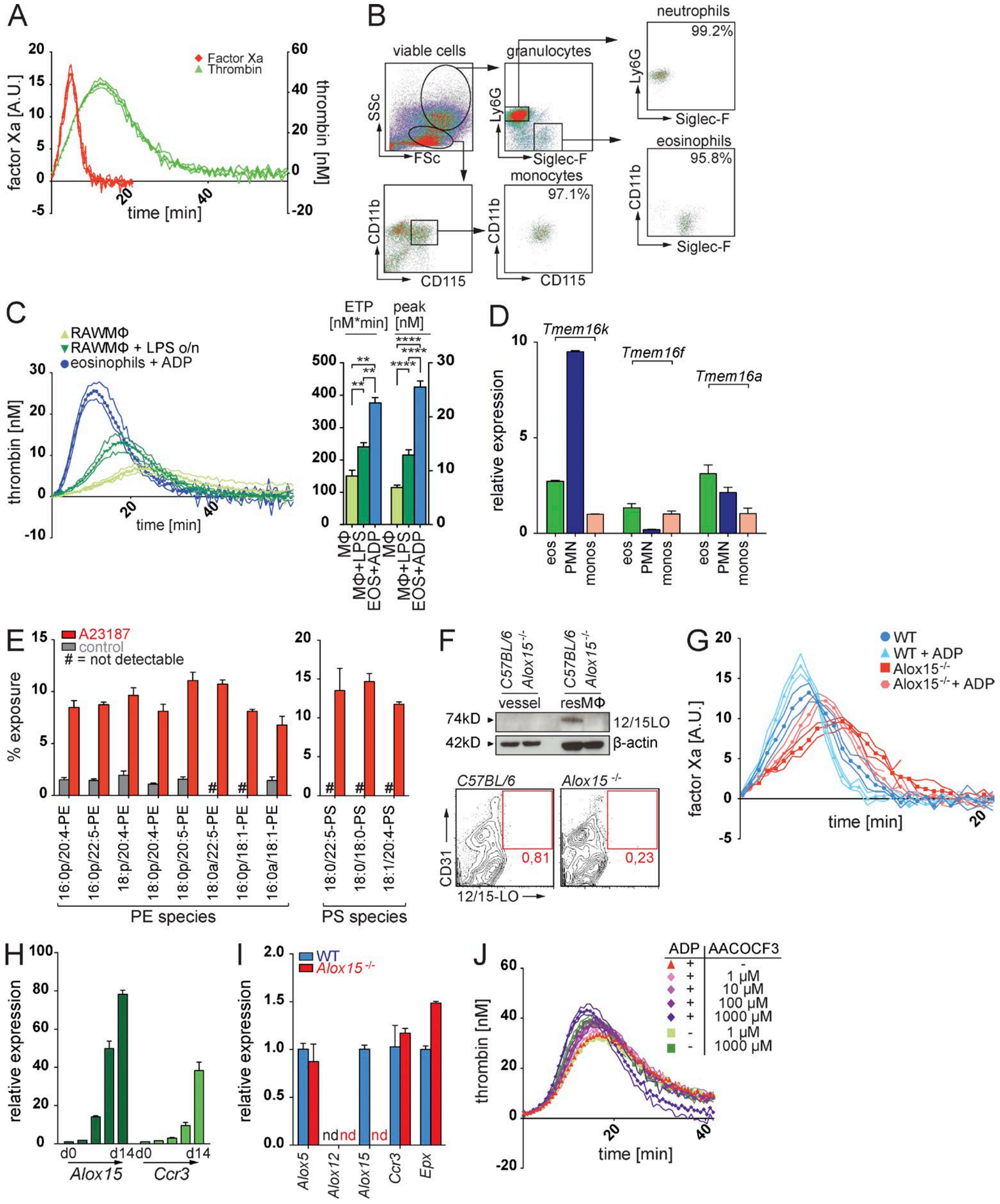

Figure S3. **Evaluation of procoagulatory activity in eosinophils.** (A) Overlap of the calibrated thrombin and FXa generation curves of mouse eosinophils. A.U., arbitrary units. (B) Gating strategy for FACS of leukocyte subpopulations isolated from bone marrow. Neutrophils were identified as CD45$^+$CD11b$^+$ (not depicted) Ly6G$^+$Siglec-F$^-$, eosinophils were identified as CD45$^+$CD11b$^+$ (not depicted) Ly6GSiglecF$^+$, and monocytes were identified as CD45$^+$ (not depicted) CD11b$^+$CD115$^+$. Percentages indicate purity of the sorted population in reanalysis. FSc, forward scatter; SSc, side scatter. (C) Calibrated thrombin generation assay with ADP-stimulated mouse eosinophils (EOS) and resting or LPS-treated RAW 264.7 macrophages (Mφ [RAWMφ]). Bar graphs show endogenous thrombin potential (ETP; nM*min) and peak of thrombin generation (peak; nM). o/n, overnight. (D) Quantitative RT-PCR analysis of *Tmem16a*, *Tmem16f*, and *Tmem16k* expression in sorted mouse leukocyte subsets. Expression was normalized to *Actb*. eos, eosinophils; monos, monocytes; PMN, neutrophils. (E) LC/MS/MS-based quantification of the exposure of the aminophospholipids PE and PS in mouse eosinophils in response to stimulation with the Ca^{2+} ionophore A23187. (F, top) Western blot analysis of 12/15-LO protein (74 kD) expression in whole-aorta lysates from WT or *Alox15*$^{-/-}$ mice and resident WT or *Alox15*$^{-/-}$ peritoneal mouse macrophages (resMφ; as positive control for 12/15-LO expression). (Bottom) Flow cytometry analysis of 12/15-LO expression in CD31$^+$ endothelial cells in a single-cell suspension generated from whole aorta isolated from WT (left) or *Alox15*$^{-/-}$ (right) mice. (G) Calibrated FXa generation curve of mouse WT eosinophils and *Alox15*$^{-/-}$ eosinophils. (H) Quantitative RT-PCR analysis of *Alox15* and *CCR3* mRNA in mouse eosinophils during 14 d of in vitro differentiation with IL-5. Expression is normalized to *Actb* and related to day 0. (I) Quantitative RT-PCR analysis of mRNA of LO isoforms (*Alox5*, *Alox12*, and *Alox15*) and eosinophil maturation markers (*Ccr3* and *Epx*) in WT and *Alox15*$^{-/-}$ mouse eosinophils. Expression is normalized to *Actb* and related to WT mouse eosinophils. (J) Calibrated thrombin generation assay with ADP-stimulated mouse eosinophils in the absence or presence of indicated concentrations of the phospholipase A2 inhibitor AACOCF3. Data are representative of at least three independent experiments. Error bars represent SEM. **, $P < 0.01$; ****, $P < 0.0001$.

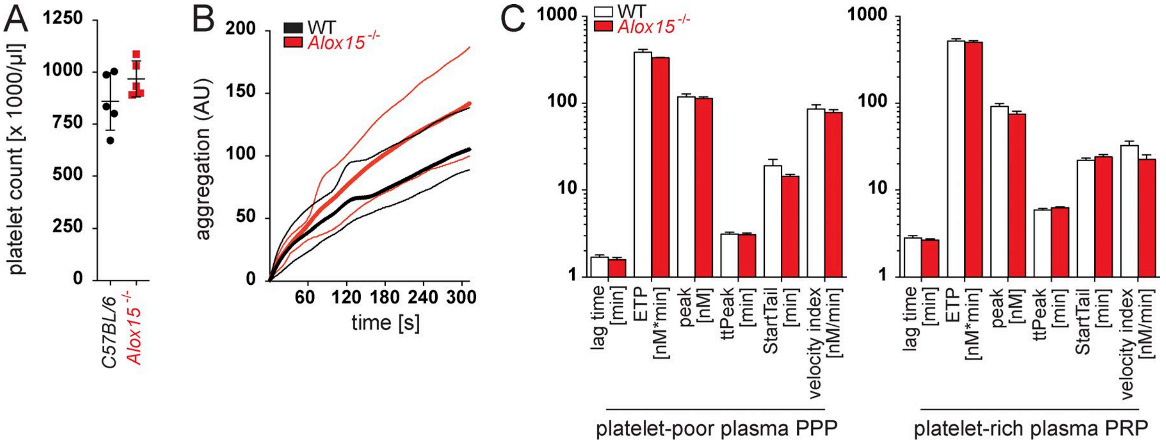

Figure S4. **Assessment of platelet function and coagulation capacity in 12/15-LO–deficient mice.** (A) Platelet counts in peripheral blood from whole blood from WT (C57BL/6) or *Alox15*$^{-/-}$ mice (n = 5 each). (B) Platelet aggregation assay with whole blood from WT mice (C57BL/6) or *Alox15*$^{-/-}$ mice (n = 4 each; thin lines represent standard error). (C) Parameters obtained from coagulometric assays with PPP (left) or PRP (right) isolated from WT (white) or *Alox15*$^{-/-}$ (red) mice. n = 4 each. Data are representative of at least three independent experiments. Error bars represent SEM. Student's t test. AU, arbitrary units; ETP, endogenous thrombin potential; ttPeak, time-to-peak.

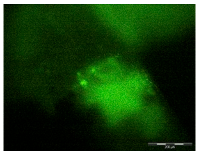

Video 1. **Initial thrombus formation within the carotid artery of a WT mouse 5 min after FeCl injury.** The playback rate is 10 frames per second.

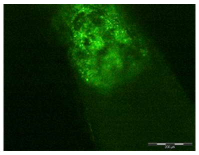

Video 2. **Stabilization of thrombus formation within the carotid artery of a WT mouse 30 min after FeCl injury.** The playback rate is 10 frames per second.

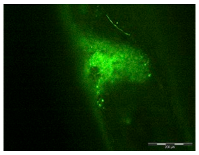

Video 3. **Initial thrombus formation within the carotid artery of a Δ*dblGATA1* mouse 5 min after FeCl injury.** The playback rate is 10 frames per second.

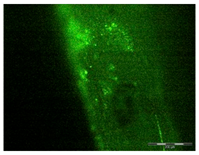

Video 4. **The dissolving of an unstable thrombus within the carotid artery of a ΔdblGATA1 mouse 30 min after FeCl injury.** The playback rate is 10 frames per second.

Table S1. **Baseline characteristics of the Bruneck Study cohort (*n* = 682)**

Characteristics	Mean ± SD or number (%)
Age (yr)	66.1 ± 10.2
Female sex (*n* [%])	354 (51.9)
Smoking, pack (yr)	12.7 ± 17.4
Body mass index (kg/m^2)	25.4 ± 4
Diabetes mellitus (*n* [%])	78 (11.4)
Hypertension (*n* [%])	382 (56.0)
HDL cholesterol (mg/dl)	57.3 ± 15.2
LDL cholesterol (mg/dl)	148.9 ± 36.7
C-reactive protein (mg/liter)[a]	1.8 (0.9–4)
Atherosclerosis score (mm)[a]	1.7 (0.0–5.7)

[a]Median (25th–75th percentile).
